# Application of Deep Learning-Based Markerless Pose Estimation in a 3D Exergame System for Knee Osteoarthritis Exercise Management: Development and Preliminary Evaluation

**DOI:** 10.3390/s26185857

**Published:** 2026-09-16

**Authors:** Xingye Cheng, Xi Gao, Wenqi Liang, Rebecca M. Meiring, Yanxin Zhang

**Affiliations:** School of Exercise, Sport and Rehabilitation Sciences, Faculty of Science, University of Auckland, Auckland 1023, New Zealand; xingye.cheng@auckland.ac.nz (X.C.); xi.gao@auckland.ac.nz (X.G.); wenqi.liang@auckland.ac.nz (W.L.); rebecca.meiring@auckland.ac.nz (R.M.M.)

**Keywords:** pose estimation algorithm, 3D exergame, rehabilitation, knee osteoarthritis

## Abstract

Home-based exercise management for people with knee osteoarthritis (KOA) is often limited by insufficient movement supervision, individualization, and progression support. This study described the development and preliminary evaluation of a KOA-specific 3D exergame exercise management system. The system combined markerless motion analysis using a conventional RGB camera with biomechanical analysis, concurrent avatar-based guidance, task-specific feedback, adaptive difficulty adjustment, pain monitoring, therapist override, and training records. Exercise tasks addressed lower-limb strength, balance, range of motion, and toe-in or toe-out gait retraining. Five physiotherapists conducted a preliminary evaluation of the prototype through technology-acceptance and usability questionnaires and semi-structured interviews. They reported generally favorable perceptions and identified clinically relevant exercise content, low sensing burden, comprehensive feedback, and training-progress monitoring as potential strengths. Concerns included fall risk during demanding tasks, limited individualization of some parameters, and insufficient safeguards for unsupervised use. Selected TDPT-derived kinematic measures were also compared with synchronized marker-based motion capture using a public dataset comprising 18 squat cycles from six participants. Agreement varied by measure: waveform associations were strong for functional hip flexion, knee flexion, trunk inclination, and frontal-plane hip abduction/adduction, whereas absolute errors ranged from 6.59° to 20.00° and the foot-orientation proxy showed negligible correlation. These findings provide preliminary clinician-informed and task-specific technical evidence for the prototype but do not establish patient usability or clinical effectiveness. Further technical validation, safety refinement, and supervised evaluation with people with KOA are required before routine or independent home use can be considered.

## 1. Introduction

Knee osteoarthritis (KOA) is one of the most common musculoskeletal conditions affecting older adults and is associated with pain, reduced lower-limb function, and limitations in daily activities and work participation [1,2]. Exercise for the management of KOA is effective for reducing knee pain, enhancing the quality of life, and improving physical function among people with KOA [3,4]. Evidence shows that exercise can improve muscle strength, stabilize the affected joint, inhibit inflammation, slow the degradation of cartilage, and prevent the loss of subchondral bone [5]. Exercise is commonly prescribed and initially instructed by therapists, who assess patients, teach appropriate movements, and adjust exercise intensity according to symptoms and functional capacity [6]. However, the long-term management of KOA often requires patients to continue exercising outside clinical settings, particularly at home. Regular clinic-based supervision is difficult to sustain because it can impose substantial financial, time, and travel burdens, especially for older adults, people in remote areas, and individuals with limited mobility [7,8]. Therefore, home-based exercise has gained increasing recognition as a practical complement to outpatient care for people with KOA [9,10,11]. It may reduce the need for frequent clinic visits and transportation burden while supporting ongoing symptom management and functional improvement [12]. However, without continued supervision from therapists, home-based exercise often lacks adequate movement guidance, timely adjustment, and adherence support [13]. These limitations highlight the need for accessible home-based approaches that can make exercise more engaging while providing objective movement monitoring and feedback.

Exergames offer a potential approach to addressing the limitations of home-based rehabilitation by converting therapeutic movements into interactive game tasks. By incorporating game objectives, scores, and immediate feedback, exergames may make repetitive exercise more engaging and less monotonous, thereby supporting enjoyment, motivation, and continued participation [14,15]. To translate physical movements into game interaction, exergame systems are commonly combined with portable motion-capture technologies, including wearable sensors [16,17], force sensors [18,19], and depth cameras [20]. These technologies can capture joint kinematics, segment displacement, balance-related measures, and spatiotemporal characteristics. The resulting data can be used to control avatars and virtual objects, assess exercise performance, generate corrective feedback, record training outcomes, and support remote review by therapists. Recent intelligent sensing research has combined flexible-sensor signals and visual data with fine-tuned multimodal large language models to support state recognition, abnormality detection, and interpretable feedback [21]. Combined with symptom reporting and clinician access to performance data, sensing-enabled exergames may support remote monitoring of home-based rehabilitation and inform subsequent exercise adjustment [22,23]. Furthermore, some systems present rehabilitation tasks within interactive computer-generated 3D environments including immersive or non-immersive virtual reality (VR) [24,25]. These environments can provide movement visualization, spatial interaction, task goals, and immediate feedback, thereby supporting more controlled and task-specific practice [26,27]. Additionally, 3D exergames can simulate activities of daily living, potentially increasing engagement and supporting the transfer of training to functional tasks [28]. Emerging evidence suggests that 3D exergame-based interventions have the potential to improve pain, physical function, balance, and movement-related outcomes in people with KOA [29,30,31], while demonstrating favorable feasibility and acceptability [32,33].

Currently, several systems have combined exergames with portable motion-capture technologies to support KOA or lower-limb rehabilitation. Jarungvittayakon et al. used two wearable inertial measurement units to provide visual feedback during home-based quadriceps exercise for people with KOA [22]. Wang et al. combined Azure Kinect with clinician-defined exercise prescriptions and personalized KOA exergames [23], while Ling et al. used Kinect-based full-body tracking, avatars, and therapist-configurable difficulty for lower-limb rehabilitation following hip replacement [34]. Although these systems demonstrate the value of movement-responsive exercise and game-based feedback, wearable sensors and depth cameras require additional equipment, sensor placement or calibration, potentially increasing the cost and setup burden of routine or home-based rehabilitation. Deep-learning-based monocular pose-estimation algorithms (PEAs) using conventional RGB video offer a lower-equipment alternative. By tracking anatomical keypoints over time, monocular PEA can generate movement trajectories and estimate 3D human pose without wearable sensors or markers [35,36,37], providing a more accessible option for at-home movement assessment and exercise guidance [38]. Wang et al. used Mediapipe BlazePose with a conventional camera to recognize prescribed knee exercises, control multiple exergames, and report task completion, joint-angle trajectories, pain, and fatigue for clinician review [39]. Zhu et al. developed a computer-vision application that delivered personalized graded exercise and immediate feedback for people with KOA [40], while Biebl et al. used a smartphone camera to detect movement deviations and provide audiovisual corrective cues for people with knee or hip osteoarthritis [41]. Among the closely related systems reviewed here, none combined multiple forms of movement feedback with baseline- and pain-informed difficulty adjustment within a KOA-specific exergame framework. This is important because knee alignment, loading-related strategies, compensatory movements, pain response, and exercise tolerance all influence safe exercise execution and long-term adherence in KOA rehabilitation [42,43,44]. In addition, FPA-based gait retraining has largely been investigated as a separate biomechanical intervention in laboratory settings, commonly using marker-based optical motion capture, instrumented treadmills or force plates, and feedback displays to quantify changes in FPA and knee loading [45,46,47], but it has rarely been integrated with strengthening and balance exercises within a single exergame system. Given the growing emphasis on scalable home-based exercise management, these limitations highlight the need for a KOA-specific system that moves beyond generic exercise delivery and incorporates clinically informed features for condition-relevant exercise guidance and progression.

Thus, this study aimed to describe the development of an early-stage KOA-specific 3D exergame-based exercise management system that integrates monocular PEA for motion analysis from conventional RGB video with condition-specific exercise design principles. A preliminary evaluation was subsequently conducted with physiotherapists to examine their perceptions of the prototype’s usability and technology acceptance and to identify the perceived strengths and limitations of each exergame. In addition, selected TDPT-derived kinematic measures were compared with synchronized marker-based motion-capture data from a publicly available dataset to provide preliminary evidence of their measurement accuracy.

## 2. System Design and Development

### 2.1. Identification of KOA-Specific Exercise Design Principles

The present 3D exergame system was developed on the basis of condition-specific exercise principles (as shown in Table 1) rather than as a generic lower-limb exercise platform. Given that exercise for KOA needs to account for pain response, functional limitations, movement quality, and gradual progression, it was necessary to incorporate principles specifically relevant to KOA into the system design. To achieve this, KOA-specific exercise design principles were derived from existing clinical recommendations, and were subsequently used to guide the development of the system.

### 2.2. System Overview

**Figure 1:**
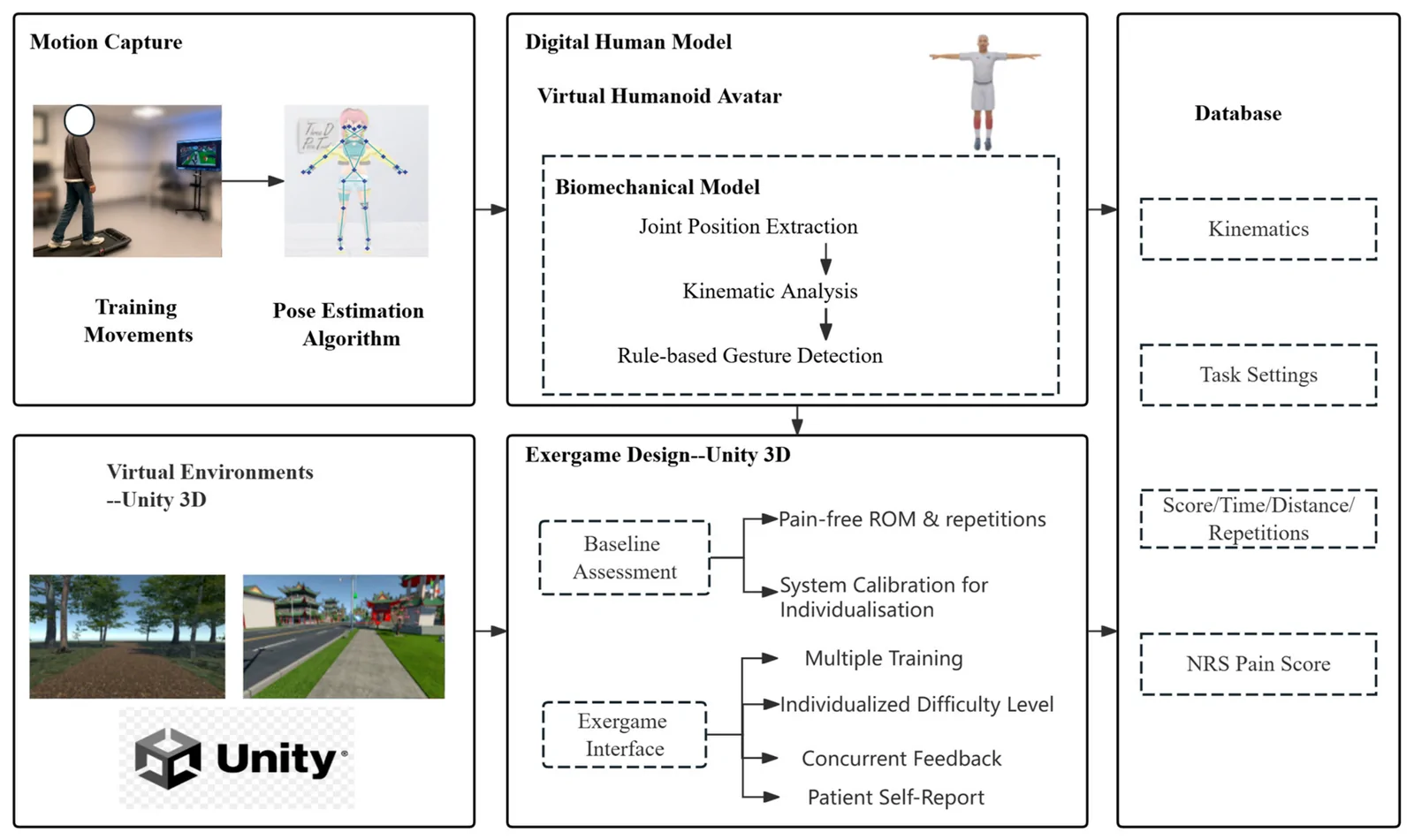
Architecture and data flow of the system. Monocular RGB video is processed by TDPT-Barracuda to estimate three-dimensional keypoints, which drive the humanoid avatar and biomechanical analysis. Baseline assessment initializes exercise parameters, the exergames provide movement feedback, and kinematic, task, performance, and pain data are stored in the database. KOA, knee osteoarthritis; RGB, red–green–blue; TDPT, ThreeDPoseTracker.

The architecture of the system is illustrated in Figure 1. The system was implemented as a screen-based, non-immersive 3D exergame in Unity 2019.4.40f1 (Unity Technologies, San Francisco, CA, USA) and ran on a Dell Precision 3571 laptop (Dell Technologies Inc., Round Rock, TX, USA) equipped with an Intel Core i7-12700H processor (Intel Corporation, Santa Clara, CA, USA), 16 GB RAM, and an NVIDIA RTX A1000 Laptop GPU (NVIDIA Corporation, Santa Clara, CA, USA) with 4 GB video memory. The application was displayed on an external television. Motion was captured using the laptop’s integrated HD RGB camera, which supported video capture at 1280 × 720 pixels and 30 frames/s. The gait-retraining task additionally required a portable treadmill. During use, the user should be positioned approximately 2 m in front of and directly facing the camera. This system integrates five core modules: motion capture, a digital human model incorporating a biomechanical model, virtual environments, exergame design, and a database. The workflow of the proposed system proceeds as follows. User movements are first captured by the motion capture module and processed using a PEA to obtain joint-level position data. These data are mapped to a virtual humanoid avatar and analyzed by a biomechanical model to extract kinematic parameters and detect relevant movement patterns. The processed information is then integrated into the Unity-based exergame, where an initial baseline assessment identifies pain-free range of motion and repetition capacity to initialize individualized training parameters. During training, concurrent movement feedback, defined as feedback presented during task execution rather than after completion of the exercise set or session [51], is provided through the avatar using visual and auditory feedback. After each completed set, self-reported pain perception is collected. Finally, kinematic data, task settings, performance metrics, and self-reported pain are recorded in the database for subsequent analysis and progress tracking.

The TDPT pose-estimation image in the Motion Capture module is reproduced from the TDPT SDK for Unity website, © Digital Standard Co., Ltd., cropped and used with permission.

### 2.3. Motion Capture System

A markerless 3D human PEA, ThreeDPoseTracker (TDPT)-Barracuda for Unity (Digital Standard Co., Ltd., Osaka, Japan) [52], is adopted to identify and track the key joint points from a monocular RGB camera. TDPT Barracuda was selected as a lower-equipment approach to movement analysis because it does not require wearable sensors or laboratory-based motion-capture equipment and is compact and convenient for use in non-laboratory settings. For each video frame, TDPT Barracuda estimated body-centered 3D keypoint coordinates relative to the abdominal region with positive X pointed toward the participant’s right, positive Y pointed upward, and positive Z pointed away from the camera. The upper and lower body were normalized separately using the navel-to-head-center and navel-to-ankle lengths, respectively [52], producing relative, non-metric coordinates that could not be interpreted directly in physical units. A TDPT model-coordinate unit refers to one unit in the relative, non-metric Pos3D coordinate system returned by TDPT-Barracuda. No confidence threshold or exclusion rule was applied; all predicted coordinates were retained, no keypoints were designated as missing, and no temporal interpolation was performed. The coordinates were processed using a fourth-order zero-phase Butterworth low-pass filter with a 6-Hz cut-off frequency and were subsequently used to drive the humanoid avatar and support movement analysis. Joint angles were calculated as the angles between body-segment vectors formed by the relevant keypoints, while task-specific events were detected from relative positions, distances, movement directions, and displacements between consecutive frames.

### 2.4. Digital Human Model

#### 2.4.1. Humanoid Avatar

To enhance the sense of presence and immersion in the virtual environment, motivate active participation in the exergame, and guide users in making appropriate movement adjustments, a digital human model (i.e., an avatar) was incorporated into the system. The humanoid avatar was created using Mixamo (Adobe Inc., San Jose, CA, USA), where a generic human mesh was automatically rigged with a standard skeletal structure and configured according to the Unity Humanoid Avatar specification. The avatar skeleton was then mapped to the joint hierarchy provided by the TDPT Barracuda body tracking system, enabling representation of 46 degrees of freedom. Joint data captured by the TDPT Barracuda were continuously streamed into Unity and applied to the corresponding avatar joints via a custom C# script, allowing the avatar to mirror the users’ real-world movements during task execution. This avatar-based movement mirroring facilitates intuitive movement quality feedback by visually reflecting the movements of the target limbs through the user’s avatar.

#### 2.4.2. Biomechanical Model

A biomechanical model was developed from the skeleton in Visual Studio 2019 (Microsoft Corporation, Redmond, WA, USA) to extract and process the user’s three-dimensional position data collected by the TDPT Barracuda. In this model, target movements such as lateral lunges, squats, hip abductions, and toe-in/out gait patterns are defined by their biomechanical features, where assessment of exercise performance is achieved through the calculation of body-segment vector relationships and spatial displacement. The event-detection thresholds were selected iteratively during internal prototype testing by comparing detected events with visually observed movements and adjusting the values to reduce missed or duplicate detections.

##### Lateral Lunge Detection

Lateral-lunge events were detected from the coordinates of the bilateral ankle and hip keypoints. After a 3-s standing calibration, the midpoint between the left and right hip keypoints was recorded as the baseline pelvis position. At each time frame, the detector calculated the three-dimensional inter-foot distance, lateral displacement of the hip midpoint from baseline, and displacement of both feet from the preceding update. A right lunge was registered when the inter-foot distance exceeded 50 TDPT model-coordinate units, the hip midpoint moved to the right of baseline, both foot displacements were below 5 model-coordinate units per update, and the cooldown was inactive; mirrored conditions detected a left lunge. Following detection, the cooldown remained active until the inter-foot distance decreased below 30 model-coordinate units. These thresholds were empirically selected for event detection.

##### Heel Strike Detection

Left and right step events were detected independently from the filtered TDPT Pos3D foot keypoints. After a 3-s standing calibration, the vertical coordinate of each foot keypoint was recorded as its floor-height baseline. At each time frame, foot height was defined as the vertical component of its Pos3D coordinate, while displacement was calculated as the three-dimensional Euclidean distance from its position in the preceding update. An event was registered when the maximum foot height reached since the previous event exceeded the current height by at least 0.05 TDPT model-coordinate units, the foot returned to within 0.01 units of its baseline, the normalized displacement vector had a downward dot product greater than 0.4, and displacement exceeded 1 model-coordinate unit per update. A 1-s debounce was then applied and the maximum height was reset.

##### Hip Abduction Detection

Hip-abduction posture was represented by the pelvis-referenced inter-leg angle, defined as the unsigned three-dimensional angle between the vectors from the pelvis point to the left and right feet. This measure was used as a proxy rather than an anatomical hip-abduction angle. The posture was assessed once when the approaching template reached the avatar. Foot height was defined as the vertical coordinate of the corresponding TDPT-driven avatar foot position. The required foot had to be higher than the opposite foot, with equal heights classified as incorrect. User and template angles were calculated between their respective left and right pelvis-to-foot vectors. A match was registered when the absolute angular difference was less than 10°.

##### Squat Detection

Squat onset and completion were detected from the vertical position of the hip. After a 3-s standing calibration, the hip height was recorded as the baseline. At each time frame, vertical displacement was calculated as the difference between the current and preceding hip heights. Squat onset was detected when the avatar was in the standing state, hip height decreased by more than 0.1 TDPT model-coordinate units below baseline, and vertical displacement was negative. This changed the state to squatting and incremented the repetition count. Squat completion was detected when hip height returned to or exceeded baseline while vertical displacement was positive, after which the state returned to standing.

Following detection of the task-specific movement events, filtered TDPT keypoints were used to derive keypoint-based functional kinematic measures. Hip, knee, and ankle angles were calculated from the sagittal-plane projections of the trunk–thigh, thigh–shank, and shank–foot segment vectors, respectively. Hip abduction was calculated as the signed frontal-plane angle between the hip-to-knee vector and a downward body reference defined from the shoulder midpoint to the hip midpoint. At each detected step event, the ankle-to-toe vector was projected onto the horizontal XZ plane by removing its vertical component. FPA was then calculated as the signed angle between the projected foot vector and the positive *Z*-axis, which represented the predefined direction of progression. Mirrored sign rules were applied to the two feet, with positive values representing toe-in and negative values representing toe-out. These measures were treated as functional proxies rather than anatomical joint angles. These parameters were used to provide knowledge of performance (KP) feedback to users through the rule-based algorithm by comparing the calculated data with the predetermined threshold (e.g., the target ROM).

### 2.5. Virtual Environments Development

In total, four virtual scenes were developed and integrated into different exergame tasks to enhance user engagement and adherence. Environmental assets, including terrain elements and interactive objects (e.g., trees, fruits, and pathways), were sourced from the Unity Asset Store and imported into the Unity project. These assets were then customized within Unity by adjusting geometric and lighting properties via the Inspector panel, and interactive behaviors, animations, and sound effects were implemented using C# scripts to support task-specific interactions and concurrent feedback.

### 2.6. Exergame Design

#### 2.6.1. Learning Phase

In traditional clinical practice, the therapists usually ask users first to perform a target rehabilitation movement to identify the range of motion that can be achieved and then design rehabilitation program based on this baseline. So, a learning phase was adopted in the system. During the learning phase, users were instructed to perform the squat, lateral-lunge, and hip-abduction tasks within a comfortable, pain-limited range. Before each movement test, on-screen text explained the 0–10 Numeric Rating Scale (NRS), where 0 represented “no pain” and 10 represented “the worst pain imaginable.” Users were instructed to perform as many repetitions as comfortably possible while maintaining their perceived pain at no more than 2 on the NRS and to stop the test if their pain exceeded this threshold. The system recorded the maximum number of repetitions completed and the maximum task-specific movement amplitude achieved within this pain threshold.

Three preset difficulty levels were provided, with default training volumes of 8, 10, and 12 repetitions per set for the Easy, Medium, and Hard levels, respectively. Based on the repetition capacity measured during the learning phase, users completing fewer than 8 repetitions were assigned to the Easy level, those completing 8–11 repetitions were assigned to the Medium level, and those completing 12 or more repetitions were assigned to the Hard level. The measured movement amplitude was used to individualize the in-game movement target for each exercise: maximum knee-flexion angle for the squat, lateral distance between the feet for the lateral lunge, and the angle between the two lower limbs for hip abduction. If a physiotherapist manually specified the exercise parameters, the therapist-defined values overrode the preset and baseline-derived settings.

#### 2.6.2. Exergame Task Content

**Figure 2:**
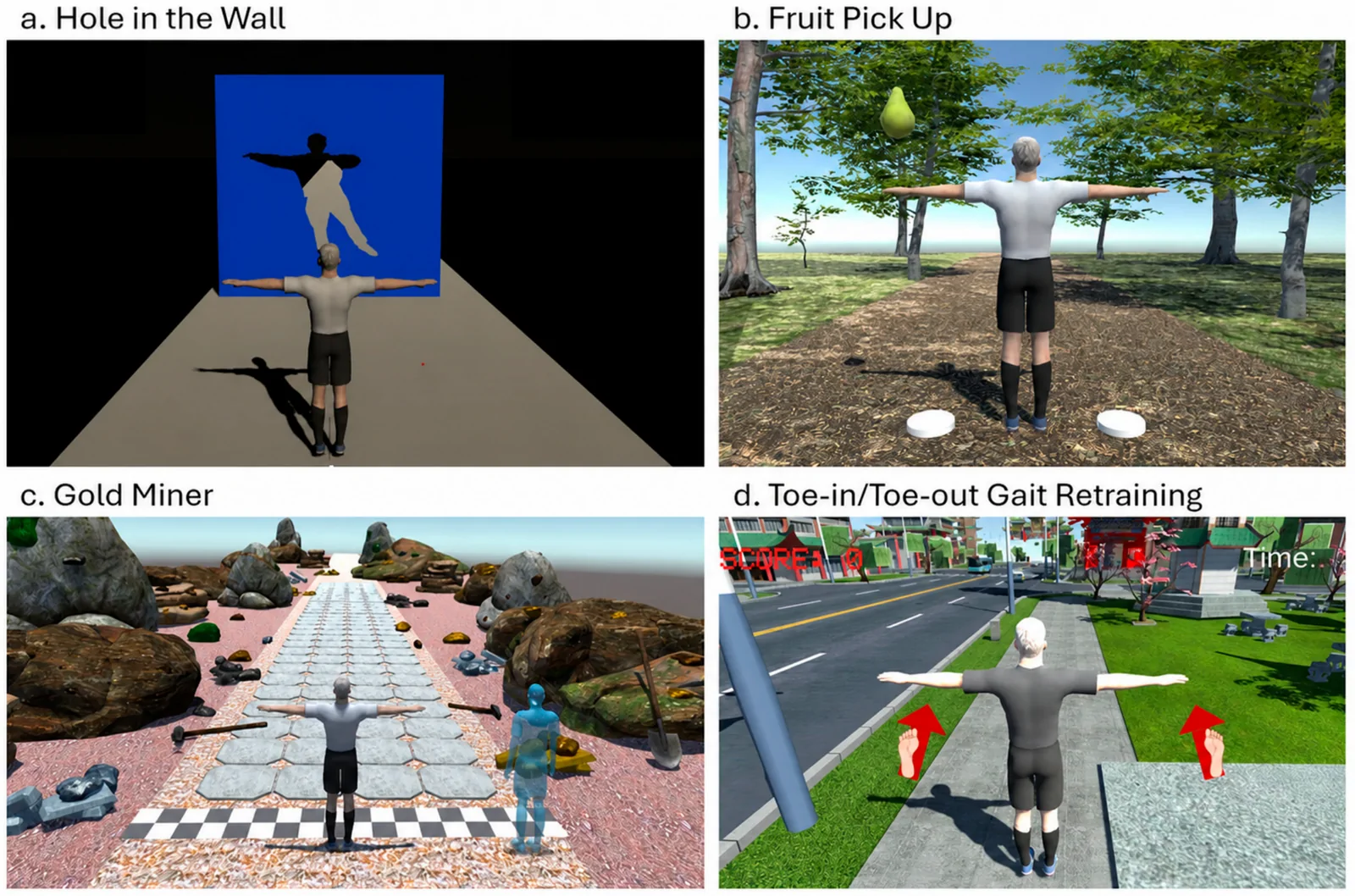
Interfaces of the four exergames. (**a**) Hole in the Wall for hip-abduction posture matching; (**b**) Fruit Pick Up for lateral-lunge training; (**c**) Gold Miner for squat training; and (**d**) toe-in/toe-out gait retraining with foot-progression-angle feedback.

Based on evidence supporting the effectiveness of both functional training and gait retraining in people with KOA, the system was designed to target these two components separately. Functional training focuses on improving lower-limb strength, balance, and active range of motion through multi-joint movements, whereas gait retraining aims to reduce knee joint loading and pain using gait modification strategies shown to be effective in this population [46,53]. Details of the exergames are presented in Table 2, and the exergame interface is illustrated in Figure 2.

#### 2.6.3. Game Flow

To align the session structure with clinical practice, the arrangement of the training session was informed by selected principles of NEMEX-TJR [50]. We retained its three-part structure of warm-up, exercise, and cool-down, together with graded exercise difficulty and an emphasis on lower-limb strength, postural control, balance, and functional movement. The original cycling warm-up and circuit program were replaced by a video-guided dynamic warm-up, screen-based exergames, and gait retraining. The task order, sets, and training volumes are summarized in Table 2. Each set lasted approximately 1 min for the three exergames and 2 min for gait retraining, with a rest period of approximately the same duration as the preceding set. Each session ended with video-guided lower-extremity stretching and lasted approximately 30–35 min.

Clinically, pain is an essential self-reported parameter in the rehabilitation process of people with KOA, and all rehabilitation movements should be performed without pain or with mild pain [50,54]. The movement difficulty, including the range of motion, training intensity, and movement repetitions, is typically adjusted according to patients’ Visual Analogue Scale score, graded from 0 to 10, in which 0 is “no pain” and 10 is “pain as bad as it could be” [54]. So, the prototype incorporated an 11-point NRS to inform individualized adjustment of exercise difficulty based on the user’s reported pain. Specifically, users are asked to report their pain after each completed set by clicking the button on an NRS-based 0–10 pain rating scale. The specific rules governing how the reported NRS score affects difficulty adjustment and task termination are described in Section 2.6.4.

#### 2.6.4. Two-Stage Adaptive Difficulty Adjustment

**Figure 3:**
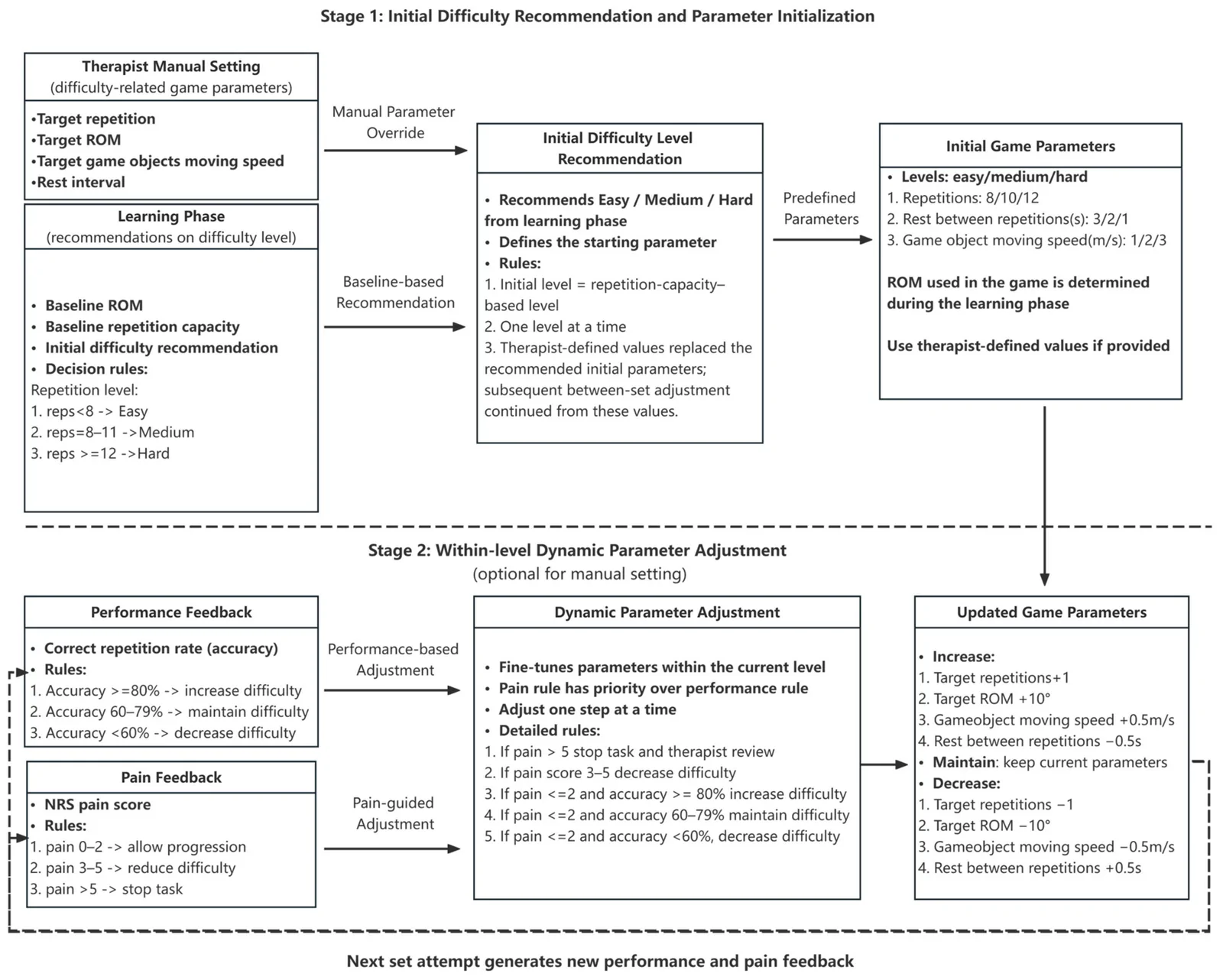
Two-stage difficulty-adjustment framework comprising baseline-informed initialization and rule-based between-set adjustment: Gold Miner Example. Stage 1 inputs comprise the repetition capacity and pain-limited ROM measured during the Learning Phase, together with any parameters entered manually by the physiotherapist. Repetition capacity determines the recommended initial difficulty level, while the pain-limited ROM defines the individualized movement target. Stage 2 inputs comprise the current game parameters, correct-repetition rate, and post-set NRS score. Pain takes priority over performance: NRS > 5 terminates the task and requires physiotherapist review; NRS 3–5 decreases difficulty; and, when NRS ≤ 2, correct-repetition rates of ≥80%, 60–79%, and <60% result in an increase, maintenance, and decrease, respectively. The output is an updated parameter set for the subsequent set, comprising target repetitions, target ROM, game-object speed, and rest between repetitions. Solid arrows indicate the flow from assessment and feedback inputs to parameter selection, while the return arrow indicates that the updated parameters become the current settings for the subsequent set. Physiotherapist-entered settings override system-generated recommendations. ROM, range of motion; NRS, Numeric Rating Scale.

Figure 3 presents the two-stage difficulty-adjustment framework using the Gold Miner squat game as an example. In Stage 1, repetition capacity measured during the Learning Phase was used to recommend an initial difficulty level, with preset targets of 8, 10, and 12 repetitions within the recommended 8–12 repetition range [50,55]. The target ROM was individualized from the maximum achieved with NRS ≤ 2. Therapist-entered parameters replaced the system recommendation and became the current settings, after which automatic between-set adjustment continued. Although the repetition range and pain-response categories were evidence-informed [50], the difficulty-level repetition boundaries and correct-repetition-rate thresholds were predefined prototype settings and remain unvalidated. In Stage 2, a correct repetition required both the individualized movement target and the corresponding game objective to be achieved. For Gold Miner, the absolute difference between the measured knee-flexion angle and the individualized target had to be within 10°, and the object had to be successfully collected. The correct-repetition rate was calculated as the percentage of attempted repetitions classified as correct. Immediately after each set and before the next set began, the user entered a NRS pain score. Pain took priority over performance [48], and the resulting decision thresholds are summarized in Figure 3. When a one-step change was triggered, all applicable parameters were updated simultaneously using the increments shown in Figure 3. Adjustments were applied only to the subsequent set. The current prototype did not enforce explicit numerical upper or lower parameter limits; progression was instead constrained by the pain rules and physiotherapist oversight. The same structure was applied to the lateral-lunge and hip-abduction games using task-specific movement targets and success criteria. For Fruit Pick Up, the absolute difference between the measured lateral step distance and the individualized target established during the Learning Phase had to be within 10 cm, and the fruit had to be successfully caught. For Hole in the Wall, the absolute difference between the measured pelvis-referenced inter-leg angle and the individualized target had to be within 10°, and the performed posture had to match the wall template. When difficulty increased or decreased, the target lateral foot distance changed by 10 cm for Fruit Pick Up and the target inter-leg angle by 10° for Hole in the Wall. For both games, target repetitions, game-object speed, and the rest interval between repetitions were adjusted using the same increments as in Gold Miner: ±1 repetition, ±0.5 m/s, and ±0.5 s, respectively. All parameters remained unchanged when difficulty was maintained. The Pseudocode for the Gold Miner Game is provided in Supplementary Material S1.

#### 2.6.5. Feedback Design

**Figure 4:**
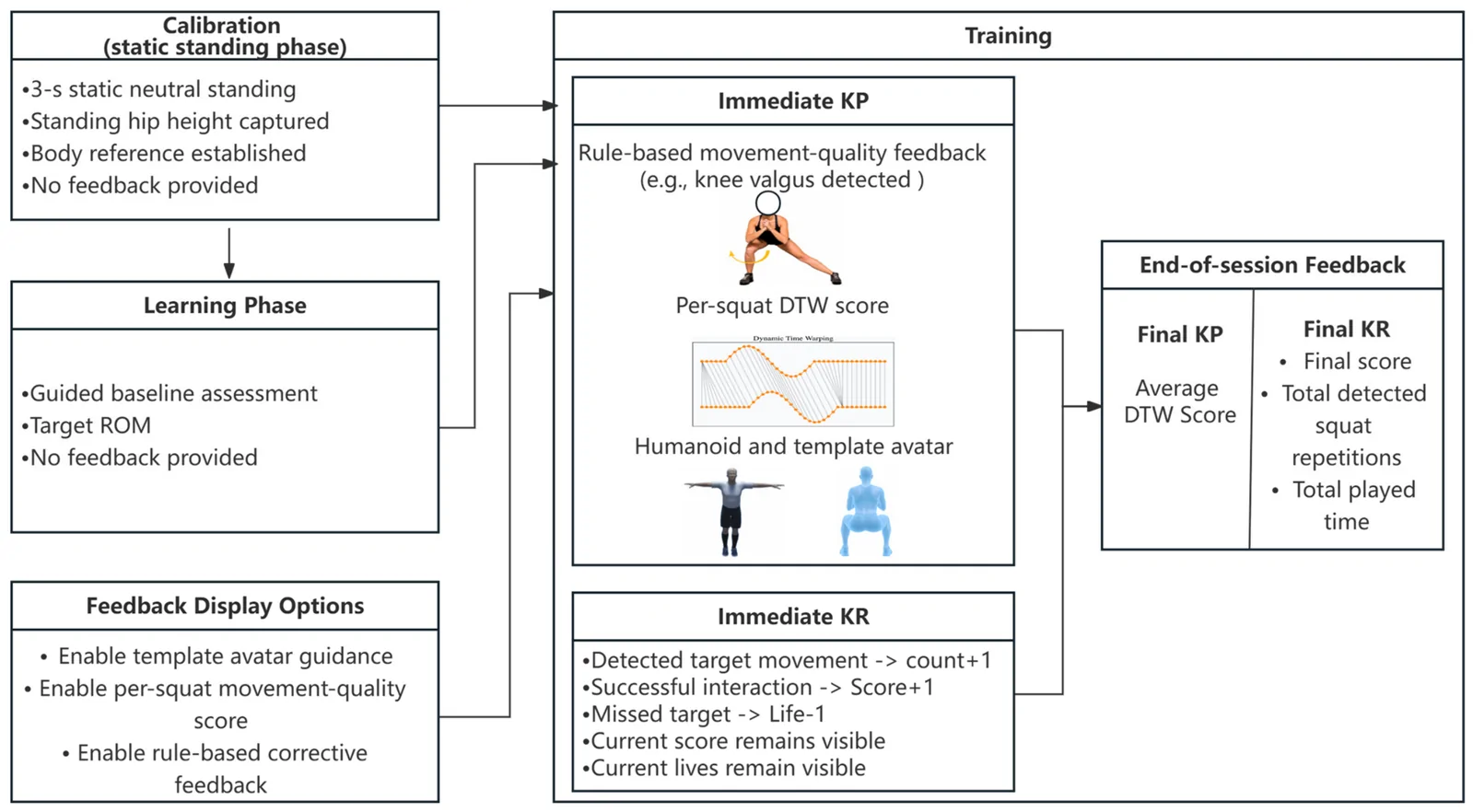
Multi-level feedback framework illustrated using the squat game. A 3-s static-standing calibration is performed before the Learning Phase baseline assessment and repeated before game training. This establishes a neutral body reference, including standing hip height, for detecting movement onset, completion, and return to the starting posture. No feedback is provided during calibration or baseline assessment. The Learning Phase determines an individualized target ROM, which is subsequently used for performance assessment and rule-based feedback during training. KP is provided through the user avatar and three independently selectable components: template-avatar guidance, rule-based corrective cues, and a post-repetition DTW-based movement-similarity score. KR is generated from gameplay events: detection of the target movement increases the repetition count, a successful interaction increases the game score, and a missed interaction reduces the remaining lives. At the end of the session, the mean DTW-based movement-similarity score is reported as summary KP, while the final game score, number of detected repetitions, and total training time constitute summary KR. DTW, dynamic time warping; KP, knowledge of performance; KR, knowledge of results; ROM, range of motion.

To align with clinical reasoning and motor learning principles, the proposed system implemented a multi-level feedback design. Following calibration and guided baseline assessment, feedback during training was structured as concurrent KP and knowledge of results (KR), and an end-of-session summary (Figure 4). The template avatar, post-repetition DTW-based movement-similarity score, and rule-based corrective feedback could be enabled independently.

Therapists routinely demonstrate the target movement when a patient performs it incorrectly or is unsure how to proceed. To reproduce this guidance, an expert performance of each movement was recorded as a standard motion template and rendered in Unity as a semi-transparent template avatar beside the user’s avatar (as shown in Figure 4), giving a continuous visual reference during task execution. To provide users with an overall indication of how closely each squat matched the template movement, dynamic time warping (DTW) distance was used to calculate a movement-similarity score after each completed repetition. DTW is a time-series comparison method that identifies an optimal nonlinear alignment between two sequences and calculates their accumulated distance along the alignment path. Its main advantage is that sequences of different lengths can be compared without requiring frame-by-frame temporal correspondence [56]. This is particularly relevant to exercise assessment because repetitions of the same movement may be performed at different speeds and durations while retaining a similar movement pattern. DTW has therefore been used in previous studies to compare exercise trajectories and derive movement-performance scores [57,58,59]. For each of the three non-gait exergames, a task-specific reference movement was recorded in advance from a physiotherapist with experience in KOA rehabilitation. The movement was captured using the same TDPT processing pipeline, and the bilateral hip, knee, and ankle angle trajectories were retained as the reference template. DTW compared the user’s and reference trajectories using time-series data based on Equation (1).

(1) $$DTW\left(P,T\right)=\underset{W}{\min}\sum_{\left(i,j\in W\right)}d(p_{i},t_{j})$$

where P and T denote the joint angle time series of the participant and the reference template, respectively; W is a valid warping path; (i,j)∈W represents aligned time indices; and $d(p_{i},t_{j})$ is the point-wise Euclidean distance between joint angles. The resulting distance was converted into a bounded movement similarity score using the standing-reference normalization described in [58] using Equation (2). Static standing represented the absence of the target movement and provided a consistent lower-performance anchor. This score reflects similarity to the selected reference trajectory rather than a clinically validated measure of movement quality.

(2) $$Movement\;similarity\;score=max\left(0,1-\frac{DTW\left(P,T\right)}{DTW\left(T,\;S\right)}\right)\times 100\;$$

where P, T, and S  denote the participant, reference, and static standing trajectories, respectively. $DTW\left(T,\;S\right)$ was interpreted as the deviation from the standing reference. The score ranged from 0 to 100, with 100 indicating an exact reference match and 0 assigned when $DTW\left(P,T\right)\geq DTW\left(T,S\right)$. The score was calculated only when $DTW\left(T,\;S\right)$ > 0.

**Figure 5:**
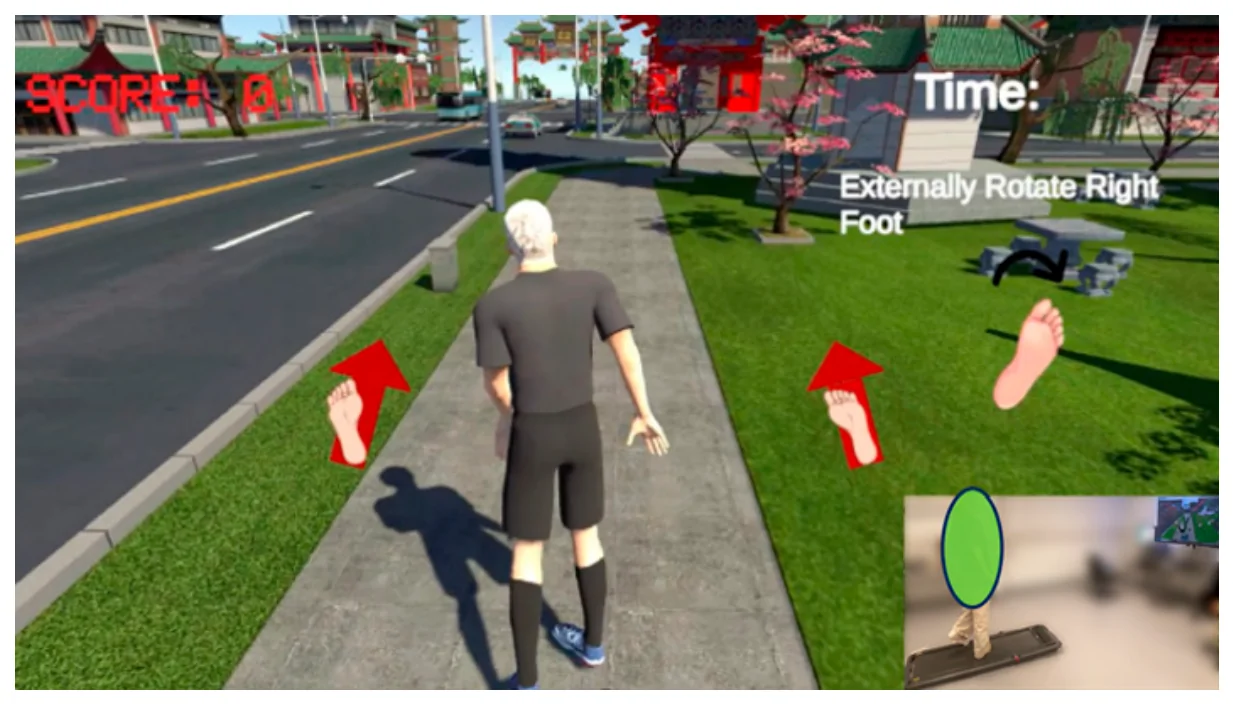
Example of arrow-based visual feedback during the toe-in gait-retraining task.

The resulting score was displayed immediately after each repetition. In parallel, rule-based corrective feedback was generated from a biomechanical model. Rule-based corrective feedback used predefined task-specific thresholds. For both squats in Gold Miner and lateral lunges in Fruit Pick Up, knee valgus was flagged when the medial knee–ankle displacement along the *X*-axis exceeded 20 TDPT model-coordinate units on either side. Insufficient forward trunk inclination was flagged when the trunk angle relative to the positive *Y*-axis, after applying a fixed 10° offset, was ≤20°. For hip-abduction posture matching, the participant–template angle difference had to be <10°; otherwise, directional leg-position cues were provided. Detected deviations triggered concise visual and auditory instructions, such as cues for insufficient ROM, knee valgus, or toe-in or toe-out positioning, as illustrated in Figure 5. KR was derived directly from gameplay events, with successful and missed interactions updating the score and remaining lives, respectively. At the end of each session, the average DTW score and overall task outcomes were summarized. This framework distinguished guidance and feedback on movement execution from information about task outcomes, while allowing the displayed feedback components to be tailored to each user.

## 3. Evaluation Methods

### 3.1. Physiotherapist Preliminary Evaluation

#### 3.1.1. Participants and Recruitment

A preliminary physiotherapist-perspective evaluation was conducted to explore perceptions of the prototype’s technology acceptance and usability. Eligible participants were physiotherapists with at least three years of experience in KOA rehabilitation. No additional exclusion criteria were specified. Participants were recruited through purposive sampling from the research team’s professional and clinical networks. Ethical approval was granted by the Northern B Health and Disability Ethics Committee (Reference Number: 2024 EXP 19133), and all participants provided written informed consent.

Five physiotherapists were recruited from hospital rehabilitation departments or physiotherapy clinics. They had a mean total clinical experience of 7.0 ± 3.2 years, a mean KOA rehabilitation experience of 6.7 ± 3.8 years, and managed approximately 19.0 ± 4.2 people with KOA per week. Two participants had previous experience with digital rehabilitation technologies: one with an exergame for fall prevention in older adults and the other with a VR exergame for gait retraining in people with stroke.

#### 3.1.2. Evaluation Procedure

Each physiotherapist attended one individual session lasting approximately 60 min, including approximately 40 min of system use. Sessions were conducted using the laptop, integrated RGB camera, and external television described in Section 2.2, while the gait-retraining component additionally used the portable treadmill. The same researcher conducted all sessions and first introduced the system’s purpose and workflow.

Five components were evaluated in a fixed order: the Learning Phase, Gold Miner, Fruit Pick Up, Hole in the Wall, and toe-in/out gait-retraining exergames. Before each component, the researcher explained its function, required movements, operation, how the correct-repetition rate was calculated, and how post-set pain and accuracy determined progression or regression of the next set, then guided the physiotherapist through one practice trial. After this familiarization, the physiotherapist configured and operated the component while the same healthy researcher without KOA acted as the exercise user. Difficulty levels were selected by the physiotherapists during operation rather than following a standardized sequence, allowing them to explore different settings. During the Learning Phase, the healthy researcher performed the movements used to generate the baseline targets. Different simulated NRS scores were subsequently entered after completed sets so that the physiotherapists could observe the corresponding pain-informed adjustments. The baseline- and pain-informed functions were therefore demonstrated using a healthy exercise user and simulated pain inputs; their clinical appropriateness, safety, and responsiveness to actual symptoms were not evaluated in people with KOA.

After evaluating all components, physiotherapists’ perceptions of technology acceptance and usability were assessed. In this study, technology acceptance referred to participants’ attitudes toward the system and willingness to adopt it in practice, whereas usability referred to the extent to which the system could be used effectively, efficiently, and satisfactorily to achieve specific goals [60]. Technology acceptance was assessed using an adapted Technology Acceptance Model (TAM) questionnaire [61], covering perceived usefulness, perceived ease of use, attitude, and intention to use. Items were rated on a 5-point Likert scale, with mean scores above 4 indicating high acceptance [60,62]. The wording was contextualized for this study by referring specifically to the exercise management system and framing its usefulness, operation, and intended adoption from the perspective of physiotherapists involved in KOA rehabilitation. These adaptations were made because the original generic technology wording did not reflect the clinical context or the physiotherapists’ role in operating and recommending the system. Usability was assessed using the 10-item System Usability Scale (SUS) [63], which uses a 5-point response scale to produce an overall score from 0 to 100, with scores above 80.3 interpreted as high satisfaction in ease of use and overall user experience [28]. After the questionnaires, the semi-structured interview was conducted to collect more detailed feedback on their overall impressions, perceived benefits, concerns, and suggestions for improvement using the interview outline provided in Supplementary Material S2. All interviews were audio-recorded, lasted between 15 and 20 min, and were transcribed into written form. A descriptive qualitative approach was used to analyze the interview data by one researcher. The researcher read the transcripts repeatedly and summarized the main meaning of each relevant statement while retaining its link to the original response and participant identifier. These summaries were organized according to the interview guide, namely each exergame and the overall system experience. Conceptually similar summaries across participants were consolidated at the reporting stage to reduce repetition. The findings were subsequently presented as perceived strengths and limitations, supported by representative quotations from the participants.

### 3.2. Validation of TDPT-Derived Kinematic Measures Using a Publicly Available Motion-Capture Dataset

Validation was conducted using a publicly available dataset containing synchronized RGB video and marker-based motion-capture data.

#### 3.2.1. Dataset and Reference Measurements

This study used the publicly available OpenCap LabValidation_withVideos dataset [64] and included only the normal squat trial (squats1) from Session 0. Synchronized Cam2 videos and marker-based kinematic data were analyzed. Videos were recorded using an iPhone 12 Pro (Apple Inc., Cupertino, CA, USA) at 60 Hz with a resolution of 720 × 1280 pixels. Cam2 was selected because its frontal view most closely resembled the camera arrangement used in the prototype. Reference marker trajectories were recorded at 100 Hz using an eight-camera Motion Analysis system (Motion Analysis Corporation, Rohnert Park, CA, USA) with 31 anatomical and 20 segment-tracking markers. Six participants were included in the analysis. For each participant, three complete squat cycles from the same body side were retained, resulting in 18 cycles in total. Cycle boundaries were identified from the OpenSim (Stanford University, Stanford, CA, USA) inverse-kinematics knee-flexion signal: local maxima represented the lowest squat position, while the adjacent local minima in the standing position defined the beginning and end of each cycle.

#### 3.2.2. Data Processing

To ensure a methodologically comparable analysis, the marker-based hip, knee, and ankle joint centers were transformed frame by frame into the ground coordinate system using OpenSim PointKinematics [65]. The right and left hip joint centers were defined as the origins of the corresponding hip joint frames in each participant-specific scaled OpenSim model. Knee and ankle joint centers were defined as the midpoints between the corresponding medial and lateral anatomical marker locations, while shoulder and toe marker trajectories were used to derive trunk and foot orientations. The synchronized Cam2 videos were processed using TDPT-Barracuda to obtain three-dimensional coordinates of the bilateral shoulders, hips, knees, ankles, and toes at 60 Hz. Pelvis-referenced coordinate frames were constructed separately for the marker-based and TDPT data using identical definitions.

Joint and segment angles were calculated for both systems using the same operational definitions and sign conventions, as summarized in Table 3. Angles were calculated at each system’s native sampling frequency and filtered using a fourth-order zero-phase Butterworth low-pass filter with a 6-Hz cut-off frequency. Marker-based angles were linearly interpolated from 100 Hz to the corresponding TDPT video timestamps. Each squat cycle was subsequently normalized to 101 points for waveform comparison, without temporal warping.

#### 3.2.3. Accuracy Measures and Statistical Analysis

Agreement between TDPT-Barracuda and marker-based motion capture was evaluated using mean absolute error (MAE), Pearson’s correlation coefficient (r), and the inter-protocol coefficient of multiple correlation (CMC). For each of the 18 squat cycles from six participants, marker-based angles were interpolated to the TDPT timestamps, and MAE and r were calculated from the paired filtered samples. MAE quantified the magnitude of the angular differences, whereas r assessed the linear association between the two trajectories. Cycle-level MAE and r values were summarized as mean ± standard deviation. Correlations were interpreted as negligible (|r| < 0.10), weak (0.10–0.39), moderate (0.40–0.69), strong (0.70–0.89), or very strong (≥0.90) [66]. Inter-protocol CMC was calculated from the 101-point time-normalized trajectories to assess overall waveform similarity across repeated cycles [67]. CMC values were interpreted as excellent (0.95–1.00), very good (0.85–0.94), good (0.75–0.84), moderate (0.60–0.74), or poor (0.00–0.59). A negative CMC radicand was reported as not calculable rather than assigned a value of zero.

## 4. Results

### 4.1. Physiotherapist Preliminary Evaluation

Among the five participating physiotherapists, mean TAM domain scores ranged from 4.47 to 4.69 out of 5, and the mean SUS score was 81.50 ± 4.54. As shown in Table 4, these results suggest generally positive perceptions of the system, including perceived usefulness, ease of use, attitudes toward the system, and intention to use it in practice. The SUS score indicated favorable physiotherapist-rated usability of the prototype. These findings describe the physiotherapists’ perceptions following their preliminary evaluation of the prototype. An example of the deidentified questionnaire data is provided in Supplementary Material S3.

Physiotherapist interviews provided further explanation of the questionnaire findings. Participants perceived several design features as potentially relevant to KOA rehabilitation, including the coverage of strength, balance, and gait exercises, markerless camera-based capture, feedback, difficulty settings, pain reporting, and training records. However, they also identified safety concerns at higher difficulty levels, limited safeguards for unsupervised use, the ability of clinician-facing manual controls to override system-recommended settings in the absence of role-based access restrictions, and game-specific issues in scoring, timing, feedback accuracy, and individualization. These findings are summarized in Table 5.

### 4.2. Agreement Between TDPT- and Reference-Based Kinematic Measures

The results are presented in Table 6. Functional hip flexion and knee flexion demonstrated very strong correlations between TDPT-Barracuda and marker-based motion capture (r = 0.945 and 0.923, respectively), with very good waveform similarity (CMC = 0.905 and 0.928). Trunk forward lean and frontal-plane functional hip abduction/adduction showed strong correlations (r = 0.882 and 0.884) and good waveform similarity (CMC = 0.804 and 0.828). Ankle dorsiflexion/plantarflexion showed a moderate correlation and moderate waveform similarity (r = 0.414, CMC = 0.686). The lowest MAE was observed for frontal-plane functional hip abduction/adduction (6.59 ± 2.85°), whereas functional hip flexion produced the largest MAE (20.00 ± 4.99°). Pelvis-referenced foot orientation showed negligible correlation (r = −0.077), and its CMC was not calculable.

## 5. Discussion

This study developed and preliminarily evaluated a KOA-specific 3D exergame system combining monocular markerless motion analysis with condition-specific exercise design, pain-informed adjustment, and therapist control. Physiotherapists reported generally favorable usability and technology acceptance, while identifying concerns regarding safety and unsupervised use. Dataset-based validation against marker-based motion capture showed that TDPT captured the trajectories of several kinematic measures, although absolute accuracy varied across variables. These findings support further development of the prototype but remain preliminary because the evaluation involved only five physiotherapists and the technical validation did not include concurrent testing in people with KOA.

The favorable Technology Acceptance Model (TAM) domain scores and System Usability Scale (SUS) score were broadly consistent with preliminary evaluations of other rehabilitation exergames. Jiao et al. reported physiotherapist-rated SUS scores of 76–85 across six post-stroke treadmill exergames, with TAM domain scores of at least 4/5 among physiotherapists and patients [28]. Similarly, the KneeBright biofeedback exergame achieved a mean SUS score of 79, suggesting generally favorable usability, and a TAM score of 3.4/5, indicating moderate acceptance [68]. Although these findings show a similar positive pattern, direct comparison is limited by differences in participants, technologies, exposure periods, and questionnaires. In the present study, the ratings may reflect favorable perceptions of the system’s usefulness, ease of use, and intention to use, while the SUS score suggested generally positive usability. Given the small sample size (n = 5), these findings should be interpreted as preliminary descriptive evidence. The interviews also identified gaps between the prototype and routine clinical practice. The physiotherapists participating in this study noted that exercise selection should be tailored to each patient’s condition and functional ability. They suggested that the current exercise levels may not accommodate all patients, highlighting the need for further evaluation with people with KOA. Feedback delivery also requires further refinement. Although the template avatar, movement-similarity score, and corrective cues can be enabled separately, presenting several forms of feedback simultaneously may overload some users. In clinical practice, physiotherapists typically prioritize particular movement cues and provide them at appropriate stages of training. Further work should therefore examine how feedback type, amount, and timing can be tailored to the user and exercise context. More broadly, these concerns regarding physical support, balance, and physiotherapist oversight also limit the current system’s suitability for home deployment. Although the three stationary exergames require only the camera-based setup and suitable exercise space, gait retraining additionally requires a treadmill with handrails and an emergency stop. Before testing with people with KOA, the system also requires eligibility and environmental safety checks, prompts for stable support, and stopping criteria for pain, dizziness, instability, loss of balance, or tracking failure. Its feasibility, usability, and safety should first be evaluated under physiotherapist supervision before individual components are considered for monitored home use.

Many digital rehabilitation systems for KOA and related lower-limb conditions rely on wearable sensors, force sensors, or depth cameras [16,23,39,69]. In contrast, the present platform used a standard RGB camera and markerless PEA, requiring less specialized equipment and a simpler setup than conventional motion-capture systems [70]. Physiotherapists also identified this lower sensing burden as a practical advantage for clinical use and as a feature that could facilitate future evaluation of the three stationary exergames in home settings. However, this reduction in sensing burden entails a trade-off in measurement accuracy. The better agreement for frontal-plane hip abduction/adduction likely reflects the frontal camera view, whereas sagittal-plane angles relied more heavily on inferred depth and were therefore more susceptible to occlusion and depth ambiguity [71]. Almeida et al. similarly reported greater hip-flexion error during deep squatting with a frontal monocular camera [72], whereas smaller sagittal-plane errors have been reported using a lateral camera view [73]. The strong waveform agreement but larger absolute errors observed here suggest that TDPT may be more suitable for tracking movement patterns and relative changes than for precise absolute angle measurement. Such pattern tracking may still be useful for identifying repetitions and larger deviations during exergame training, although accuracy remains important for threshold-based feedback. Physiotherapists have been shown to consistently detect changes of 12° or more in slow, single-plane movements [74]. The observed MAEs of several measures were within or close to this magnitude. Although the dataset-based validation did not reproduce real-time prototype use, the squat task corresponded to the movement implemented in Gold Miner and therefore provided preliminary, task-relevant evidence of TDPT’s kinematic performance. These indirect comparisons do not establish equivalence with therapist observation but suggest that TDPT may support feedback on larger movement deviations, while precise assessment and small-angle clinical decisions require validated measurement methods. Accordingly, the current single-camera TDPT pipeline should not be regarded as a substitute for clinical goniometry or marker-based optical motion capture when precise joint-angle measurement is required. Although limited variation during squatting and sensitivity to ankle and toe keypoint errors may have contributed to the negligible correlation, the present findings do not support reliable FPA measurement with the current configuration. Because the stationary foot-orientation proxy is not equivalent to FPA during walking, the gait-retraining feedback requires separate walking-based validation before clinical use. Although the current TDPT implementation showed limited accuracy for some measures, monocular pose-estimation methods continue to improve, with recent approaches incorporating biomechanical constraints and pose optimization achieving greater kinematic accuracy [75]. Because the pose-estimation layer can be replaced without changing the downstream game logic, future versions could incorporate improved models following task-specific and real-time validation.

At the framework level, the system was organized around rehabilitation principles relevant to KOA rather than game mechanics alone [49,50]. The two-stage framework first used Learning Phase results to match the initial ROM and repetition targets to individual capacity, and then used the correct-repetition rate and post-set NRS score to adjust the subsequent set. Pain took priority over performance in these decisions, reflecting the clinical emphasis on symptom response during KOA exercise progression [50]. This differs from previous KOA exergame and VR studies, in which pain was generally treated as an outcome measure or recorded for adverse-event monitoring rather than used to inform exercise adjustment [34]. Although the repetition range and pain-response categories were literature-informed and ROM targets were measured individually, other operational settings, including difficulty-level boundaries, correct-repetition-rate cut-offs, and between-set adjustment increments, were predefined during prototype development. Their reliability and clinical appropriateness have not been evaluated in people with KOA and therefore require validation and refinement through supervised patient testing before being used to guide exercise progression in this population.

In addition, unlike published KOA VR/exergame systems that have focused mainly on strengthening and mobility exercises, the present framework incorporated gait retraining, which may reduce knee loading and pain [46,47,53]. The inclusion of a gait retraining task expanded the training content of the platform. However, a predefined FPA target was used to standardize prototype testing because baseline FPA and knee-loading responses were not assessed; it should not be interpreted as a clinical prescription. The appropriate direction and magnitude vary between individuals. Uhlrich et al. [76] selected individualized targets by testing 5° and 10° toe-in and toe-out modifications and identifying the condition that produced the greatest reduction in knee adduction moment for each participant. The improvements achieved with this personalized approach therefore do not support prescribing a single fixed target, indicating that following system iterations should instead allow patient-specific FPA targets to be selected. Moreover, measurement accuracy is also critical. Rokhmanova et al. [77] found that foot-progression-angle estimation errors resulted in inappropriate cues and slower gait learning. The current prototype derived FPA from TDPT-estimated relative 3D keypoints and depth must be inferred from a single camera view. This component could be affected by viewpoint, occlusion, and pose-estimation errors, potentially reducing feedback accuracy. Shin et al. demonstrated that combining video and IMU data may improve tracking robustness under sensor misalignment, occlusion, and video noise [78], although the additional sensors would increase the equipment and setup burden. Nevertheless, monocular PEA remains valuable for supporting exercise training, movement tracking, and feedback outside laboratory settings because it requires only a conventional RGB camera and reduces equipment costs and setup burden. Continued advances in monocular pose-estimation algorithms have the potential to further improve the accuracy of 3D keypoint estimation [75].

At the level of feedback design, the system incorporated multiple complementary feedback mechanisms, including the mirroring avatar, rule-based cues for movement errors, and a quantitative score indicating similarity to the template movement. This may have been attractive for physiotherapists because it made movement quality visible in a way that resembles demonstration and correction in face-to-face rehabilitation, rather than relying only on task completion or game score. However, the present system used explicit spatial and angular rules to evaluate movement quality. This approach is traceable and can map a violated criterion directly to concurrent corrective feedback [41,79]. Such rules may be suitable for relatively simple, clearly defined deviations, such as failure to reach a target range of motion, knee–ankle displacement exceeding a preset threshold, or insufficient trunk inclination. These thresholds were empirically selected rather than validated against biomechanical reference measurements and may not adequately assess more complex errors, such as inappropriate coordination among the knee, hip, pelvis, and trunk, deviations occurring only during a specific movement phase, or compensatory movements developing across successive repetitions. To address these limitations, deep-learning models can learn nonlinear spatial and temporal patterns from labelled skeleton sequences and may therefore be better suited to detecting multi-joint and phase-dependent errors [80], although they require representative expert-labelled data and are less readily auditable than explicit rules. In addition, Large Language Models (LLMs) have recently been used to interpret structured movement features and generate natural-language assessments and corrective feedback [81], but may produce unsupported thresholds, overconfident judgments, or inconsistent outputs. Future exergames could therefore combine explicit rules for simple movement errors with validated deep-learning models for complex multi-joint and temporal deviations, while using LLMs to communicate feedback grounded in measured kinematics. The effectiveness of these feedback approaches also depends on their timely delivery. However, end-to-end latency from physical movement to avatar updates or corrective feedback was not directly measured. Although the camera operated at 30 Hz, total latency may also include image acquisition and buffering, pose inference, filtering, feedback processing, Unity rendering, and display refresh. As latency may affect the timeliness and usefulness of feedback, future work should quantify motion-to-avatar and movement-to-cue latency using the complete prototype configuration.

Several limitations of this study should be acknowledged. First, the technical validation of the current markerless motion-analysis pipeline was limited to offline analysis of frontal-view squat videos from a public dataset and did not reproduce real-time gameplay or include people with KOA. It therefore provided preliminary evidence for selected kinematic measures but did not validate the complete motion-analysis and feedback pipeline across all exergames, particularly gait retraining. Second, the evaluation included only physiotherapists and did not assess patient usability, adherence, safety, symptom response, or functional outcomes. The system was also not tested in home environments. Accordingly, this evaluation should be interpreted solely as preliminary formative feedback from physiotherapists on the prototype’s design and perceived usability, rather than evidence of patient usability or acceptability, clinical efficacy, long-term adherence, or safety. In addition, the interview data were summarized by a single researcher without a formal codebook or independent coding, which may have increased the influence of researcher interpretation. Future studies should therefore evaluate the prototype and the pain-contingent adaptation rules with people with KOA, initially under physiotherapist supervision and subsequently in monitored home settings if safety is established, while using a more rigorous qualitative analysis procedure where appropriate. Third, the prototype used baseline assessment and post-set pain and performance feedback to adjust the subsequent repetition target. However, therapist-defined parameters could override these settings, and the manual controls lacked role-based access restrictions. The current prototype should therefore be regarded as a physiotherapist-supervised system rather than an autonomous system. Future work should validate the adaptation rules in people with KOA and implement role-based access controls before unsupervised home use.

## 6. Conclusions

This study developed an initial KOA-oriented 3D exergame system combining monocular markerless motion analysis with clinically informed exercise management. Preliminary formative feedback from five physiotherapists indicated generally favorable usability and technology acceptance, while identifying the need for supported exercise progression, better-tailored feedback delivery, and stronger safety and oversight functions. Dataset-based validation showed that TDPT captured the movement patterns of several squat-related measures, although absolute accuracy varied and gait-related FPA estimation remains to be validated. Further task-specific technical validation and prospective, supervised studies involving people with KOA are required to evaluate patient acceptability, clinical effectiveness, long-term adherence, and the safety of the pain-contingent adaptation logic before routine or home use can be considered.

## Figures and Tables

**Table 1:** KOA-Specific Exercise Design Principles and Their Theoretical Rationale.

Principles	Rationale
Baseline Assessment and Personalization [48,49]	Exercise for KOA should begin with assessment of pain and physical function. Exercise type, dose, and progression should then be tailored to the individual rather than prescribed as a fixed generic program. Tailor the exercise program to the individual based on assessment findings.
Multicomponent Exercise [48,49]	Neuromotor exercise includes various motor skills, including balance, coordination, gait, agility, proprioceptive training and activities combining neuromotor exercise, resistance exercise and flexibility
Adequate Dose and Progressive Overload [48,49,50]	Exercise should be delivered at a sufficient frequency, intensity, and duration. Exercise difficulty should be adjusted gradually according to the individual’s response and improving capability.
Pain-informed Adjustment and Safety Monitoring [49,50]	Ensure that the exercise program is tailored to the level of pain. Progress the exercise program gradually, as long as the individual does not experience significant increases in pain or discomfort.
Clear Instruction and Performance Feedback [49]	Exercise is more likely to be implemented safely and correctly when instructions are clear, the person understands how to perform the task, and feedback is provided on performance and outcomes.
Movement Quality and Neuromuscular Control [50]	Exercises should be performed with appropriate lower-limb alignment. Emphasize movement quality and neuromuscular control during functional tasks to reduce undesirable compensatory movements.

**Table 2:** Description of the exergames.

Exergames	Task	Main Aim	Description	Difficulty Levels (Adjustable Parameters)	Training Volume
Gold Miner	Squat	To improve lower-limb strength and active knee range of motion in the sagittal plane.	Squatting to collect objects placed along the path	Pre-set levels: 3 (Squat depth)	3 sets of 8–12 reps/set
Fruit Pick Up	Lateral Lunge	To improve single-leg strength, active hip range of motion, and knee stability	Performing lateral lunge to reach falling fruit on either side of the user	Pre-set levels: 3 (Lateral foot distance)	3 sets of 8–12 reps/set
Hole in the Wall	Hip Abduction	To improve single-leg balance, hip abduction strength, and proprioception	Matching hip abduction postures to pass through approaching walls	Pre-set levels: 3 (Angle between the left and right pelvis-to-foot vectors)	3 sets of 8–12 reps/set
Gait Retraining	Toe-in/Toe-out gait	Reduce the knee pain by decreasing the knee adduction moment	Toe-in/toe-out gait retraining was performed on a treadmill with arrow-based visual feedback indicating deviation from a target FPA.	Walking speed	3 sets of 2 min/set

**Table 3:** Operational definitions of the kinematic measures used for comparison.

Measure	Operational Definition	Plane/Reference	Positive Direction
Trunk forward inclination	Angle between the mid-hip-to-mid-shoulder vector and the global vertical	Sagittal plane	Forward inclination
Functional hip flexion	Signed relative angle between the trunk and thigh segment vectors	Sagittal plane	Hip flexion
Functional hip abduction	Signed angle between the hip-to-knee segment vector and the pelvis-referenced vertical axis	Frontal plane	Hip abduction
Knee flexion	Signed relative angle between the thigh and shank segment vectors	Sagittal plane	Knee flexion
Ankle dorsiflexion	Signed relative angle between the shank and ankle-to-toe segment vectors	Sagittal plane	Dorsiflexion
Pelvis-referenced toe-out angle	Signed angle between the ankle-to-toe axis and the pelvis anterior axis	Horizontal plane	Toe-out

Note: The pelvis-referenced toe-out angle was treated as a proxy for foot orientation rather than a conventional gait foot progression angle because the squat had no direction of progression and TDPT-Barracuda did not provide a heel landmark. Functional hip flexion refers to the segment-based angle used in this study rather than a three-dimensional anatomical joint angle.

**Table 4:** Rating results for the Technology Acceptance Model (TAM) and System Usability Scale (SUS) questionnaires.

TAM				SUS
Perceived Usefulness	Perceived Ease of Use	Attitude	Intention of Use	
4.51 ± 0.19 (4.22–4.67)	4.47 ± 0.24 (4.22–4.78)	4.69 ± 0.43 (4.11–5.00)	4.67 ± 0.21 (4.44–4.89)	81.50 ± 4.54 (77.50–87.50)

Note: Values are presented as mean ± SD (min–max).

**Table 5:** Physiotherapist-perspective evaluation of the exergame system.

Scope	Strengths	Limitations	Representative Quotations
Overall system experience	1. The multimodule system covers clinically relevant KOA rehabilitation domains, including strength/function, lateral control and loading, balance, and gait. 2. Gamified goals and immediate rewards may make repetitive long-term exercise more engaging and support adherence. 3. RGB sensing reduces wearable or controller burden and supports accessible clinical use with potential for home use. 4. An individual Learning Phase baseline is more appropriate than applying one universal joint-angle target. 5. Correct-repetition rate based on game-object interaction and personal-baseline attainment is more clinically meaningful than game score alone. 6. Requiring both low post-set pain and adequate movement accuracy before progression is cautious and clinically preferable to using either criterion alone. 7. Immediate visual feedback can help patients connect a movement with its recognized outcome and support early learning. 8. The prototype integrates baseline learning, instruction, game training, symptom reporting, feedback, and adaptation within a relatively complete workflow.	1. Physiotherapist assessment, prescription, pause or override authority, and visibility of automatic changes remain necessary around the game flow. 2. Task completion, baseline attainment, movement quality, symptoms, and the causes of low accuracy should be reported separately rather than compressed into one result. 3. A first-session baseline or joint-angle target may not represent a safe, appropriate movement to reinforce and therefore requires physiotherapist confirmation. 4. Uncertain RGB-tracking accuracy and reliability may affect the validity of corrective feedback, particularly for subtle knee or foot measurements. 5. Fixed pain and correct-repetition rate thresholds are useful defaults but may be too binary or unsuitable for every movement, usual pain level, or stage of rehabilitation. 6. Elevated pain and low correct-repetition rate can reflect clinically different problems and should not trigger the same progression or regression response. 7. Feedback may confuse patients or encourage dependence if it is excessive, clinically incomplete, or not tailored separately to patient and physiotherapist needs. 8. At its current stage, the system is not suitable for unsupervised use and still requires physiotherapist oversight to ensure safe and appropriate exercise.	1. Strength: “One thing I really like is that the system does not progress someone just because they achieved a high game score. It also looks at whether they reached the game target and their own baseline, and then considers that movement accuracy together with their pain.” 2. Limitation: “But I would still need to judge whether the thresholds are appropriate, whether the baseline is reliable, and which parameter should change when a patient does not meet one of the criteria. Automation could save time, but I would need to see what the system is doing and be able to change it when necessary.”
Fruit Pick Up	1. Fruit Pick Up makes lateral movement practice more engaging and may encourage loading toward the painful side. 2. Its simple external target provides a clear focus for practicing or observing lateral weight shift, stepping, and lunge-related control.	1. Fruit timing can encourage rushing or compensation, so target contact may occur without genuine weight transfer, knee control, or stable return. 2. The game lacks therapist-controlled clinical goals and does not ensure a brief hold, controlled return, and regained stability before presenting the next target.	1. Strength: “Its main strength is making lateral training more engaging and potentially encouraging loading of the painful side.” 2. Limitation: “Its limitation is that the fruit may encourage rushing and compensation.”
Gold Miner	1. Gold Miner maps directly to functional squat relevant to daily activities and quadriceps capacity. 2. Coin targets can increase engagement and give the functional movement an understandable game objective. 3. Combining coin contact, the personal squat baseline, and pain is more clinically meaningful than counting coins alone and discourages fast shallow progression.	1. Unsupported squatting is not an appropriate starting point for every patient and requires a graded sit-to-stand, chair-height, and support pathway. 2. Coin speed and timing can drive movement pace or lower correct-repetition rate even when squat range and pain are acceptable. 3. Completing the coin task and attaining the baseline angle do not by themselves demonstrate adequate overall movement quality. 4. The system does not distinguish whether low performance results from coin timing, insufficient squat range, or elevated pain, although these problems require different responses.	1. Strength: “Gold Miner is the easiest game in which to prescribe exercise dosage. Squat can be organized into clear repetitions and sets.” 2. Limitation: “Before the system automatically makes the next set easier, I would want to know which criterion the patient did not meet and why. Missing a coin may simply mean that it appeared too quickly, whereas failing to reach the baseline may indicate insufficient squat range. Pain of 2 or higher is different again because it reflects the patient’s symptom response. These problems require different actions, such as slowing the coins, adjusting the squat range, or reducing the exercise load, rather than applying the same regression to all of them.”
Hole in the Wall	1. Hole in the Wall can train single-leg loading, hip and postural control, dynamic balance, and confidence in loading the painful side. 2. Matching the body position shown by the wall opening makes dynamic-balance practice more enjoyable and engaging.	1. Moving-wall time pressure and wall-passage scoring can create fall risk or reward unstable compensation, so the task needs staged static, supported, and therapist-controlled progression.	1. Strength: “I think this game could be useful for practicing single-leg loading, hip control, and dynamic balance. It may also help patients feel more confident putting weight through the painful side.” 2. Limitation: “I can see how the game could help patients practice balance and standing on one leg. My concern is that the moving wall puts them under time pressure, which could make some patients anxious and increase their risk of falling.”
Gait Retraining FPA	1. FPA is a distinctive gait-specific module with clinical potential for selected patients. 2. Live arrows, visual displays, or simple directional cues can make current and target foot direction easier to understand than verbal instruction alone.	1. FPA retraining is not a routine or freely selected game and should be used only after gait assessment establishes an appropriate clinical indication. 2. The game lacks an individualized clinical assessment to determine the most appropriate FPA target for each patient.	1. Strength: “One feature that really stands out is the live arrows. They make it easy for patients to see which way their foot is currently pointing and how it needs to change.” 2. Limitation: “The current game asks the physiotherapist to enter an FPA target, which assumes we already know the right angle for that patient. In practice, there is no simple clinical test for this.”

**Table 6:** Agreement and waveform similarity between TDPT-Barracuda and marker-based motion capture for selected kinematic metrics.

Kinematic Metric	MAE (°) Mean ± SD	Pearson’s r Mean ± SD	CMC
Functional hip flexion	20.00 ± 4.99	0.945 ± 0.04	0.905
Knee flexion	13.62 ± 2.42	0.923 ± 0.06	0.928
Ankle dorsiflexion/plantarflexion	8.31 ± 3.21	0.414 ± 0.67	0.686
Trunk forward lean	10.25 ± 4.68	0.882 ± 0.10	0.804
Frontal-plane functional hip abduction/adduction	6.59 ± 2.85	0.884 ± 0.07	0.828
Pelvis-referenced foot orientation (FPA proxy)	7.05 ± 3.28	−0.077 ± 0.29	-

Note: The CMC for the pelvis-referenced foot-orientation proxy was not calculable because the CMC expression produced a negative value under the square root due to the limited variation of this measure during stationary squatting.

## Data Availability

The OpenCap laboratory-validation dataset used to validate the TDPT-derived kinematic measures, including synchronized RGB videos and marker-based motion-capture data, is publicly available through the OpenCap project on SimTK (*LabValidation_withVideos*): https://simtk.org/frs/?group_id=2385 (accessed on 28 August 2026). The analyzed subset comprised the *squats1* trial from Session 0 and the synchronized Cam2 video. Other data supporting the findings of this study are available from the corresponding author upon reasonable request, subject to ethical and confidentiality restrictions. Interview transcripts are not publicly available to protect participant privacy.

## Supplementary Materials

The following supporting information can be downloaded at: https://www.mdpi.com/article/10.3390/s26185857/s1, Supplementary Material S1: Pseudocode for the Gold Miner Game; Supplementary Material S2: Interview Outline for Physiotherapists; Supplementary Material S3: De-identified Questionnaire Responses from One Participant.

## Abbreviations

KOA	Knee Osteoarthritis
VR	Virtual Reality
PEA	Pose Estimation Algorithm
TDPT	ThreeDPoseTracker
KR	Knowledge of results
KP	Knowledge of performance
DTW	Dynamic Time Warping
ROM	Range of Motion
TAM	Technology Acceptance Model
SUS	System Usability Scale

## References

[1] Woolf A.D., Pfleger B. (2000). Burden of major musculoskeletal conditions. Bull. World Health Organ..

[2] Prieto-Alhambra D., Judge A., Javaid M.K., Cooper C., Diez-Perez A., Arden N.K. (2014). Incidence and risk factors for clinically diagnosed knee, hip and hand osteoarthritis: Influences of age, gender and osteoarthritis affecting other joints. Ann. Rheum. Dis..

[3] Brosseau L., Taki J., Desjardins B., Thevenot O., Fransen M., Wells G.A., Mizusaki Imoto A., Toupin-April K., Westby M., Álvarez Gallardo I.C. (2017). The Ottawa panel clinical practice guidelines for the management of knee osteoarthritis. Part two: Strengthening exercise programs. Clin. Rehabil..

[4] Fransen M., McConnell S., Harmer A.R., Van der Esch M., Simic M., Bennell K.L. (2015). Exercise for osteoarthritis of the knee: A Cochrane systematic review. Br. J. Sports Med..

[5] Zeng C.-Y., Zhang Z.-R., Tang Z.-M., Hua F.-Z. (2021). Benefits and Mechanisms of Exercise Training for Knee Osteoarthritis. Front. Physiol..

[6] Chen H., Zheng X., Huang H., Liu C., Wan Q., Shang S. (2019). The effects of a home-based exercise intervention on elderly patients with knee osteoarthritis: A quasi-experimental study. BMC Musculoskelet. Disord..

[7] Wang G., Fan Y., Wu M., Jin X., Zhang H., Yao L., Sun Y., Su B., Ma Z. (2022). An intelligent remote exergame system for knee osteoarthritis rehabilitation training. Acta Medica Mediterr..

[8] Si J., Sun L., Li Z., Zhu W., Yin W., Peng L. (2023). Effectiveness of home-based exercise interventions on pain, physical function and quality of life in individuals with knee osteoarthritis: A systematic review and meta-analysis. J. Orthop. Surg. Res..

[9] Thomas K.S., Muir K.R., Doherty M., Jones A.C., O’Reilly S.C., Bassey E.J. (2002). Home based exercise programme for knee pain and knee osteoarthritis: Randomised controlled trial. BMJ.

[10] Anwer S., Alghadir A., Brismée J.-M. (2016). Effect of Home Exercise Program in Patients with Knee Osteoarthritis: A Systematic Review and Meta-analysis. J. Geriatr. Phys. Ther..

[11] Karasavvidis T., Hirschmann M.T., Kort N.P., Terzidis I., Totlis T. (2020). Home-based management of knee osteoarthritis during COVID-19 pandemic: Literature review and evidence-based recommendations. J. Exp. Orthop..

[12] Holden M.A., Haywood K.L., Potia T.A., Gee M., McLean S. (2014). Recommendations for exercise adherence measures in musculoskeletal settings: A systematic review and consensus meeting (protocol). Syst. Rev..

[13] Campbell R. (2001). Why don’t patients do their exercises? Understanding non-compliance with physiotherapy in patients with osteoarthritis of the knee. J. Epidemiol. Community Health.

[14] Da Silva Júnior J.L.A., Biduski D., Bellei E.A., Becker O.H.C., Daroit L., Pasqualotti A., Tourinho Filho H., De Marchi A.C.B. (2021). A Bowling Exergame to Improve Functional Capacity in Older Adults: Co-Design, Development, and Testing to Compare the Progress of Playing Alone Versus Playing with Peers. JMIR Serious Games.

[15] Sheehy L., Bharadwaj L., Nissen K.A., Estey J.L. (2025). Non-Immersive Virtual Reality Exercise Can Increase Exercise in Older Adults Living in the Community and in Long-Term Care: A Randomized Controlled Trial. Clin. Interv. Aging.

[16] Chen K.-H., Chen P.-C., Liu K.-C., Chan C.-T. (2015). Wearable Sensor-Based Rehabilitation Exercise Assessment for Knee Osteoarthritis. Sensors.

[17] Plavoukou T., Kasnesis P., Contiero Syropoulou A., Papagiannis G., Stasinopoulos D., Georgoudis G. (2025). Wearable Sensor–Based Telerehabilitation Versus Conventional Physiotherapy in Knee OA: Insights from the KneE-PAD Pilot Study. Appl. Sci..

[18] Darcy R., Couri J., Newkirk K., Neagu R., Darbhe V., Jayabalan P. (2025). The impact of a sensor based platform on the outcome of medial off-loader bracing in individuals with knee osteoarthritis: A pilot randomized clinical trial. Am. J. Phys. Med. Rehabil..

[19] Xie J., Li S., Song Z., Shu L., Zeng Q., Huang G., Lin Y. (2024). Functional Monitoring of Patients with Knee Osteoarthritis Based on Multidimensional Wearable Plantar Pressure Features: Cross-Sectional Study. JMIR Aging.

[20] Antón D., Nelson M., Russell T., Goñi A., Illarramendi A. (2016). Validation of a Kinect-based telerehabilitation system with total hip replacement patients. J. Telemed. Telecare.

[21] Mao Z., Wang J., Zhang J., Ohgi J., Zheng Y., Peng Y., Zhao L., Su Q., Huang W., Xu B. (2026). Fine-tuned multimodal large language model for autonomous state cognition system of shape-recognition 6-bar tensegrity integrated with flexible sensors. Microsyst. Nanoeng..

[22] Jarungvittayakon C., Sa-ngasoongsong P., Chockchaisakul D., Bamrungchaowkasem J., Wongsak S. (2025). Effectiveness of Mobile Exergaming with Sensor-Based Visual Feedback as an Adjunct Therapy for Home-Based Quadriceps Exercise Training in Knee Osteoarthritis: A Prospective Randomized Controlled Trial. Life.

[23] Wang G., Yao L., Fan Y., Zhang H., Jin X., Tang Q., Jiang J., Su B., Ma Z. (2024). Design of an exergame system for knee osteoarthritis rehabilitation based on the exercise prescription. Multimed. Tools Appl..

[24] Soltani P., Andrade R. (2021). The Influence of Virtual Reality Head-Mounted Displays on Balance Outcomes and Training Paradigms: A Systematic Review. Front. Sports Act. Living.

[25] Faria A.L., Latorre J., Silva Cameirão M., Bermúdez i Badia S., Llorens R. (2023). Ecologically valid virtual reality-based technologies for assessment and rehabilitation of acquired brain injury: A systematic review. Front. Psychol..

[26] Peebles A.T., van der Veen S., Stamenkovic A., France C.R., Pidcoe P.E., Thomas J.S. (2022). A Virtual Reality Game Suite for Graded Rehabilitation in Patients with Low Back Pain and a High Fear of Movement: Within-Subject Comparative Study. JMIR Serious Games.

[27] Merians A.S., Poizner H., Boian R., Burdea G., Adamovich S. (2006). Sensorimotor training in a virtual reality environment: Does it improve functional recovery poststroke?. Neurorehabil Neural Repair.

[28] Jiao Y., Dajime P.F., Zhang Q., Reading S., Smith M.-C., Zhang Y. (2025). A clinically oriented VR-based treadmill training system for post-stroke rehabilitation: Integration of motor learning and control principles. Virtual Real..

[29] Özlü A., Ünver G., Tuna H.İ., Menekşeoğlu A.K. (2023). The Effect of a Virtual Reality-Mediated Gamified Rehabilitation Program on Pain, Disability, Function, and Balance in Knee Osteoarthritis: A Prospective Randomized Controlled Study. Games Health J..

[30] Oliveira L.K.R., Marques A.P., Andrade K.F.A., de Assis J.C.S., Brito A.L., Souza G.S., Callegari B. (2024). Virtual Reality in Improving Anticipatory Postural Adjustments to Step Initiation in Individuals with Knee Osteoarthritis: A Randomized Controlled Trial. Games Health J..

[31] Guede-Rojas F., Mendoza C., Rodríguez-Lagos L., Soto-Martínez A., Ulloa-Díaz D., Jorquera-Aguilera C., Carvajal-Parodi C. (2025). Effects of Non-Immersive Virtual Reality Exercise on Self-Reported Pain and Mechanical Hyperalgesia in Older Adults with Knee and Hip Osteoarthritis: A Secondary Analysis of a Randomized Controlled Trial. Medicina.

[32] Lo H.H.M., Ng M., Fong P.Y.H., Lai H.H.K., Wang B., Wong S.Y.-S., Sit R.W.S. (2024). Examining the Feasibility, Acceptability, and Preliminary Efficacy of an Immersive Virtual Reality-Assisted Lower Limb Strength Training for Knee Osteoarthritis: Mixed Methods Pilot Randomized Controlled Trial. JMIR Serious Games.

[33] Gür O., Başar S. (2023). The effect of virtual reality on pain, kinesiophobia and function in total knee arthroplasty patients: A randomized controlled trial. Knee.

[34] Ling Y., Ter Meer L.P., Yumak Z., Veltkamp R.C. (2017). Usability Test of Exercise Games Designed for Rehabilitation of Elderly Patients After Hip Replacement Surgery: Pilot Study. JMIR Serious Games.

[35] Cao Z., Hidalgo G., Simon T., Wei S.-E., Sheikh Y. (2019). OpenPose: Realtime Multi-Person 2D Pose Estimation using Part Affinity Fields. IEEE Trans. Pattern Anal. Mach. Intell..

[36] Bazarevsky V., Grishchenko I., Raveendran K., Zhu T., Zhang F., Grundmann M. (2020). BlazePose: On-device Real-time Body Pose tracking. arXiv.

[37] Salisu S., Danyaro K.U., Nasser M., Hayder I.M., Younis H.A. (2025). Review of models for estimating 3D human pose using deep learning. PeerJ Comput. Sci..

[38] Scott B., Seyres M., Philp F., Chadwick E.K., Blana D. (2022). Healthcare applications of single camera markerless motion capture: A scoping review. PeerJ.

[39] Wang G., Zhu B., Fan Y., Wu M., Wang X., Zhang H., Yao L., Sun Y., Su B., Ma Z. (2022). Design and evaluation of an exergame system to assist knee disorders patients’ rehabilitation based on gesture interaction. Health Inf. Sci. Syst..

[40] Zhu D., Zhao J., Wu T., Zhu B., Wang M., Han T. (2025). Effects of a Computer Vision–Based Exercise Application for People with Knee Osteoarthritis: Randomized Controlled Trial. JMIR Mhealth Uhealth.

[41] Biebl J.T., Rykala M., Strobel M., Bollinger P.K., Ulm B., Kraft E., Huber S., Lorenz A. (2021). App-Based Feedback for Rehabilitation Exercise Correction in Patients with Knee or Hip Osteoarthritis: Prospective Cohort Study. J. Med. Internet Res..

[42] Bennell K.L., Dobson F., Hinman R.S. (2014). Exercise in osteoarthritis: Moving from prescription to adherence. Best Pract. Res. Clin. Rheumatol..

[43] Bannuru R.R., Osani M.C., Vaysbrot E.E., Arden N.K., Bennell K., Bierma-Zeinstra S.M.A., Kraus V.B., Lohmander L.S., Abbott J.H., Bhandari M. (2019). OARSI guidelines for the non-surgical management of knee, hip, and polyarticular osteoarthritis. Osteoarthr. Cartil..

[44] van Tunen J.A.C., Dell’Isola A., Juhl C., Dekker J., Steultjens M., Thorlund J.B., Lund H. (2018). Association of malalignment, muscular dysfunction, proprioception, laxity and abnormal joint loading with tibiofemoral knee osteoarthritis—A systematic review and meta-analysis. BMC Musculoskelet. Disord..

[45] Shull P.B., Shultz R., Silder A., Dragoo J.L., Besier T.F., Cutkosky M.R., Delp S.L. (2013). Toe-in gait reduces the first peak knee adduction moment in patients with medial compartment knee osteoarthritis. J. Biomech..

[46] Uhlrich S.D., Silder A., Beaupre G.S., Shull P.B., Delp S.L. (2018). Subject-specific toe-in or toe-out gait modifications reduce the larger knee adduction moment peak more than a non-personalized approach. J. Biomech..

[47] Simic M., Wrigley T.V., Hinman R.S., Hunt M.A., Bennell K.L. (2013). Altering foot progression angle in people with medial knee osteoarthritis: The effects of varying toe-in and toe-out angles are mediated by pain and malalignment. Osteoarthr. Cartil..

[48] Moseng T., Vliet Vlieland T.P.M., Battista S., Beckwée D., Boyadzhieva V., Conaghan P.G., Costa D., Doherty M., Finney A.G., Georgiev T. (2024). EULAR recommendations for the non-pharmacological core management of hip and knee osteoarthritis: 2023 update. Ann. Rheum. Dis..

[49] Holden M.A., Metcalf B., Lawford B.J., Hinman R.S., Boyd M., Button K., Collins N.J., Cottrell E., Henrotin Y., Larsen J.B. (2023). Recommendations for the delivery of therapeutic exercise for people with knee and/or hip osteoarthritis. An international consensus study from the OARSI Rehabilitation Discussion Group. Osteoarthr. Cartil..

[50] Ageberg E., Link A., Roos E.M. (2010). Feasibility of neuromuscular training in patients with severe hip or knee OA: The individualized goal-based NEMEX-TJR training program. BMC Musculoskelet. Disord..

[51] Sigrist R., Rauter G., Riener R., Wolf P. (2013). Augmented visual, auditory, haptic, and multimodal feedback in motor learning: A review. Psychon. Bull. Rev..

[52] Aoyagi Y., Yamada S., Ueda S., Iseki C., Kondo T., Mori K., Kobayashi Y., Fukami T., Hoshimaru M., Ishikawa M. (2022). Development of Smartphone Application for Markerless Three-Dimensional Motion Capture Based on Deep Learning Model. Sensors.

[53] Richards R., van den Noort J.C., van der Esch M., Booij M.J., Harlaar J. (2018). Gait retraining using real-time feedback in patients with medial knee osteoarthritis: Feasibility and effects of a six-week gait training program. Knee.

[54] Clausen B., Holsgaard-Larsen A., Søndergaard J., Christensen R., Andriacchi T.P., Roos E.M. (2014). The effect on knee-joint load of instruction in analgesic use compared with neuromuscular exercise in patients with knee osteoarthritis: Study protocol for a randomized, single-blind, controlled trial (the EXERPHARMA trial). Trials.

[55] Levinger P., Dunn J., Bifera N., Butson M., Elias G., Hill K.D. (2017). High-speed resistance training and balance training for people with knee osteoarthritis to reduce falls risk: Study protocol for a pilot randomized controlled trial. Trials.

[56] Sakoe H., Chiba S. (1978). Dynamic programming algorithm optimization for spoken word recognition. IEEE Trans. Acoust. Speech Signal Process..

[57] Yu X., Xiong S. (2019). A Dynamic Time Warping Based Algorithm to Evaluate Kinect-Enabled Home-Based Physical Rehabilitation Exercises for Older People. Sensors.

[58] Saraee E., Gu Y., Pandit S., Tran S., Shandelman E., Singh S., Nordahl T.J., Ellis T., Betke M. ExerciseCheck: Data analytics for a remote monitoring and evaluation platform for home-based physical therapy. Proceedings of the 12th ACM International Conference on PErvasive Technologies Related to Assistive Environments.

[59] Su C.-J., Chiang C.-Y., Huang J.-Y. (2014). Kinect-enabled home-based rehabilitation system using Dynamic Time Warping and fuzzy logic. Appl. Soft Comput..

[60] Birckhead B., Khalil C., Liu X., Conovitz S., Rizzo A., Danovitch I., Bullock K., Spiegel B. (2019). Recommendations for Methodology of Virtual Reality Clinical Trials in Health Care by an International Working Group: Iterative Study. JMIR Ment. Health.

[61] Davis F.D. (1989). Perceived Usefulness, Perceived Ease of Use, and User Acceptance of Information Technology. MIS Q..

[62] Melas C.D., Zampetakis L.A., Dimopoulou A., Moustakis V. (2011). Modeling the acceptance of clinical information systems among hospital medical staff: An extended TAM model. J. Biomed. Inform..

[63] Brooke J., Jordan P.W., Thomas B., Weerdmeester B.A., McClelland I.L. (1996). SUS: A “Quick and Dirty” Usability Scale. Usability Evaluation in Industry.

[64] Uhlrich S.D., Falisse A., Kidziński Ł., Muccini J., Ko M., Chaudhari A.S., Hicks J.L., Delp S.L. (2023). OpenCap: Human movement dynamics from smartphone videos. PLoS Comput. Biol..

[65] Seth A., Hicks J.L., Uchida T.K., Habib A., Dembia C.L., Dunne J.J., Ong C.F., DeMers M.S., Rajagopal A., Millard M. (2018). OpenSim: Simulating musculoskeletal dynamics and neuromuscular control to study human and animal movement. PLoS Comput. Biol..

[66] Schober P., Boer C., Schwarte L.A. (2018). Correlation Coefficients: Appropriate Use and Interpretation. Anesth. Analg..

[67] Kadaba M.P., Ramakrishnan H.K., Wootten M.E., Gainey J., Gorton G., Cochran G.V. (1989). Repeatability of kinematic, kinetic, and electromyographic data in normal adult gait. J. Orthop. Res..

[68] Krepkovich E., Kaur M., Mangum L.C., Saliba S., Lichter M., Olowin A., Richardson N., Hart J. (2022). Feasibility of a Novel Video Game-Based Electromyography Biofeedback System in Patients with Knee Osteoarthritis. J. Sport Rehabil..

[69] Chen P.-C., Huang C.-N., Chen I.-C., Chan C.-T., Biswas J., Kobayashi H., Wong L., Abdulrazak B., Mokhtari M. (2013). A Rehabilitation Exercise Assessment System Based on Wearable Sensors for Knee Osteoarthritis. Inclusive Society: Health and Wellbeing in the Community, and Care at Home.

[70] Lam W.W.T., Tang Y.M., Fong K.N.K. (2023). A systematic review of the applications of markerless motion capture (MMC) technology for clinical measurement in rehabilitation. J. Neuroeng. Rehabil..

[71] Cheng X., Jiao Y., Meiring R.M., Sheng B., Zhang Y. (2025). Reliability and validity of current computer vision based motion capture systems in gait analysis: A systematic review. Gait Posture.

[72] de Almeida L.P., Guenka L.C., de Oliveira Felipe D., Ishii R.P., de Campos P.S., Burke T.N. (2023). Correlation between MOVA3D, a Monocular Movement Analysis System, and Qualisys Track Manager (QTM) during Lower Limb Movements in Healthy Adults: A Preliminary Study. Int. J. Environ. Res. Public Health.

[73] Stenum J., Rossi C., Roemmich R.T. (2021). Two-dimensional video-based analysis of human gait using pose estimation. PLoS Comput. Biol..

[74] Abbott E., Campbell A., Wise E., Tidman S.J., Lay B.S., Kent P. (2022). Physiotherapists could detect changes of 12 degrees or more in single-plane movement when observing forward bending, squat or hand-over-head: A cross-sectional experiment. Musculoskelet. Sci. Pract..

[75] Gilon S., Miller E.Y., Ulrich S.D. (2026). OpenCap Monocular: 3D human kinematics and musculoskeletal dynamics from a single smartphone video. arXiv.

[76] Uhlrich S.D., Mazzoli V., Silder A., Finlay A.K., Kogan F., Gold G.E., Delp S.L., Beaupre G.S., Kolesar J.A. (2025). Personalised gait retraining for medial compartment knee osteoarthritis: A randomised controlled trial. Lancet Rheumatol..

[77] Rokhmanova N., Pearl O., Kuchenbecker K.J., Halilaj E. (2024). IMU-Based Kinematics Estimation Accuracy Affects Gait Retraining Using Vibrotactile Cues. IEEE Trans. Neural Syst. Rehabil. Eng..

[78] Shin S., Li Z., Halilaj E. (2023). Markerless Motion Tracking with Noisy Video and IMU Data. IEEE Trans. Biomed. Eng..

[79] Zhao W., Reinthal M.A., Espy D.D., Luo X. (2017). Rule-Based Human Motion Tracking for Rehabilitation Exercises: Realtime Assessment, Feedback, and Guidance. IEEE Access.

[80] Marusic A., Nguyen S.M., Tapus A. (2025). Skeleton-Based Transformer for Classification of Errors and Better Feedback in Low Back Pain Physical Rehabilitation Exercises. arXiv.

[81] Tang J., Abedi A., Colella T.J.F., Khan S.S., Khan S.S., Romeo L., Abedi A. (2025). Rehabilitation Exercise Quality Assessment and Feedback Generation Using Large Language Models with Prompt Engineering. ArtifiAI for Aging Rehabilitation and Intelligent Assisted Living.

