# Supplementary material for: Application of Deep Learning-Based Markerless Pose Estimation in a 3D Exergame System for Knee Osteoarthritis Exercise Management: Development and Preliminary Evaluation
Source: Sensors (Basel). 2026 Sep 16;26(18):5857. doi: 10.3390/s26185857 (PMC13611215; doi:10.3390/s26185857)
Supplement: Supplementary file 1 [file sensors-26-05857-s001.zip › sensors-4481796-supplementary.pdf]

Supplementary Materials

S1 Pseudocode for the Gold Miner Game

SquatGameManager.cs

```
VARIABLES
    recommendedDifficulty
    initialKneeROM
    currentParameters
    nextParameters

    attemptedRepetitions = 0
    correctRepetitions = 0
    NRS PainScore

INITIALIZE GAME
    Obtain recommended difficulty and initial knee ROM
    from the Learning Phase

    IF therapist-defined settings are available
        Use therapist-defined difficulty, knee ROM,
        repetitions, object speed and rest interval
    ELSE
        Use the recommended difficulty and corresponding preset:
            Easy:   8 repetitions, speed 1, spawn interval 3 s
            Medium: 10 repetitions, speed 2, spawn interval 2 s
            Hard:   12 repetitions, speed 3, spawn interval 1 s

        Use initial knee ROM as the individualized movement target
    END IF

    Wait 2 seconds
    Apply the selected parameters
    Display the current difficulty level

    Start 3-second countdown
    Start timer and object spawning
END INITIALIZATION

DURING GAME
    Provide real-time game, movement and DTW feedback

    FOR each attempted squat
        attemptedRepetitions += 1

        IF target knee ROM is reached
           AND the corresponding object is collected
            correctRepetitions += 1
        END IF
    END FOR
END GAMEPLAY

WHEN CURRENT SET ENDS
    Stop object spawning and timer
    Display game score, squat repetitions and average DTW score
```

```
    IF attemptedRepetitions > 0
        correctRate =
            correctRepetitions / attemptedRepetitions × 100
    ELSE
        correctRate = 0
    END IF

    Collect NRS pain score
    nextParameters = currentParameters

    IF NRSPainScore > 5
        Stop the task and request therapist review

    ELSE IF NRSPainScore is between 3 and 5
        Decrease difficulty for the next set

    ELSE IF NRSPainScore <= 2
        IF correctRate >= 80%
            Increase difficulty for the next set
        ELSE IF correctRate >= 60%
            Maintain current difficulty
        ELSE
            Decrease difficulty for the next set
        END IF
    END IF

    IF difficulty is increased
        nextParameters.repetitions += 1
        nextParameters.targetKneeROM += 10 degrees
        nextParameters.objectSpeed += 0.5 Unity units/second
        nextParameters.restInterval -= 0.5 seconds

    ELSE IF difficulty is decreased
        nextParameters.repetitions -= 1
        nextParameters.targetKneeROM -= 10 degrees
        nextParameters.objectSpeed -= 0.5 Unity units/second
        nextParameters.restInterval += 0.5 seconds
    END IF

    IF therapist modifies the proposed parameters
        Replace nextParameters with therapist-defined values
    END IF

    Save nextParameters for the next set
    Save score, repetitions and NRS pain score
END SET
```

SquatDetector.cs

```
VARIABLES
    hipTransform
    baselineHipHeight
    previousHipPosition
    currentHipPosition

    squatted
    squatCount = 0

    beginSquatDisplacement = 0.1 Unity units
```

```
    displacementThreshold = 0 Unity units/update
    velocityThreshold = 0.1 Unity units/second

    hipVelocityY
    isLowest

INITIALIZATION
    wait 2 seconds
    baselineHipHeight = hipTransform.position.y

EVERY UNITY UPDATE
    currentHipPosition = hipTransform.position
    deltaY = currentHipPosition.y - previousHipPosition.y

    IF squatted == FALSE
       AND currentHipPosition.y < baselineHipHeight - 0.1
       AND deltaY < 0
    THEN
        squatted = TRUE
        squatCount = squatCount + 1
        invoke onSquatBegin
            → DTWScoreManager.StartRecord()

    END IF

    IF squatted == TRUE
       AND currentHipPosition.y >= baselineHipHeight
       AND deltaY > 0
    THEN
        squatted = FALSE
        invoke onSquatEnd
             → DTWScoreManager.StopRecord()
             → DTWScoreManager.CalculateDTW()
    END IF

    hipVelocityY =
        (currentHipPosition.y - previousHipPosition.y)
        / Time.deltaTime

    IF squatted == TRUE
       AND hipVelocityY > 0.1
    THEN
        isLowest = TRUE
    ELSE
        isLowest = FALSE
    END IF

    previousHipPosition = currentHipPosition
END UPDATE

FUNCTION GetSquatCount
    RETURN squatCount
END FUNCTION
```

JointAngleCalculator.cs

```
DURING EACH UNITY UPDATE WHILE DTW RECORDING IS ACTIVE

    obtain TDPT-Barracuda Pos3D coordinates for:
        bilateral hips
```

```
        bilateral knees
        bilateral ankles
        bilateral toes
        nose
        upper abdomen

    project the required vectors onto the sagittal Y-Z plane
        by setting the X component to zero

    calculate:
        right knee flexion angle
        left knee flexion angle
        right ankle flexion angle
        left ankle flexion angle
        right hip flexion angle
        left hip flexion angle

    append the six angles as one time point
        to the participant trajectory
END
```

DTWScoreManager.cs

```
templateStandingDistance =
    SimpleDTW.ComputeNormalizedDTW(template, standing)

participantTemplateDistance =
    SimpleDTW.ComputeNormalizedDTW(participant, template)

epsilon = a small positive value

IF templateStandingDistance <= epsilon
    // Additional safeguard because the equation is undefined
    // when DTW(template, standing) equals zero.
    IF participantTemplateDistance <= epsilon
        similarityScore = 100
    ELSE
        similarityScore = 0
    END IF
ELSE
    similarityScore =
        MAX(
            0,
            1 - participantTemplateDistance
                / templateStandingDistance
        ) × 100
END IF

displayedScore = ROUND(similarityScore)
display displayedScore
append displayedScore to session score list
END
```

SimpleDTW.cs

```
FUNCTION ComputeNormalizedDTW(sequenceX, sequenceY)

    nx = number of time points in sequenceX
    ny = number of time points in sequenceY
```

```
CREATE accumulatedCost[nx + 1, ny + 1]
SET all values to positive infinity
accumulatedCost[0, 0] = 0

FOR i = 1 TO nx
    FOR j = 1 TO ny

        localDistance = 0

        FOR dimension = 1 TO 6
            difference =
                sequenceX[i - 1, dimension]
                - sequenceY[j - 1, dimension]

            localDistance += difference²
        END FOR

        localDistance = SQUARE_ROOT(localDistance)

        accumulatedCost[i, j] =
            localDistance
            + MINIMUM(
                accumulatedCost[i - 1, j],
                accumulatedCost[i, j - 1],
                accumulatedCost[i - 1, j - 1]
              )
    END FOR
END FOR

totalDistance = accumulatedCost[nx, ny]

// Backtrack the optimal warping path
i = nx
j = ny
warpingPathLength = 0

WHILE i > 0 AND j > 0
    warpingPathLength += 1

    predecessor =
        ARGMIN(
            accumulatedCost[i - 1, j],
            accumulatedCost[i, j - 1],
            accumulatedCost[i - 1, j - 1]
        )

    IF predecessor is diagonal
        i -= 1
        j -= 1
    ELSE IF predecessor is vertical
        i -= 1
    ELSE
        j -= 1
    END IF
END WHILE

IF warpingPathLength == 0
    RETURN positive infinity
END IF
```

```
    normalizedDistance =
        totalDistance / warpingPathLength

    RETURN normalizedDistance
END FUNCTION
```

SquatReminderTrigger.cs

```
VARIABLES
    templateAvatarAnimator

WHEN A MOVING GAME OBJECT ENTERS THE REMINDER TRIGGER

    IF the entering object contains a Projectile component
        trigger the "Squat" animation
            on the template avatar
    END IF

END
```

SquatReminder.cs

```
VARIABLES
    BarracudaRunner
    SquatDetector

    targetSquatDepth                 // From Learning Phase or therapist
input

    valgusThreshold = 20             // TDPT Pos3D model-coordinate units
    trunkAngleOffset = 15 degrees
    minimumTrunkFlexion = 20 degrees
    feedbackDuration = 2 seconds

    assessingSquat = FALSE
    peakSquatDepth = 0

    rightValgusAtPeak = FALSE
    leftValgusAtPeak = FALSE
    trunkFlexionAtPeak = 0

    feedbackText
    rightValgusImage
    leftValgusImage
    trunkFlexionImage
    squatDepthImage
    excellentAudio
    feedbackTimer


INITIALIZATION
    disable all feedback images

    connect SquatDetector.onSquatBegin
        to StartSquatAssessment

    connect SquatDetector.onSquatEnd
```

```
        to EvaluateSquat
END


FUNCTION StartSquatAssessment
    assessingSquat = TRUE
    peakSquatDepth = 0

    rightValgusAtPeak = FALSE
    leftValgusAtPeak = FALSE
    trunkFlexionAtPeak = 0
END


EVERY UNITY UPDATE

    IF feedbackTimer > 0
        feedbackTimer -= Time.deltaTime
    ELSE
        disable all feedback images
        clear feedbackText
    END IF

    IF assessingSquat == FALSE
        RETURN
    END IF

    keypoints = BarracudaRunner.GetKeyPoint()

    rightHip = keypoints[right hip].Pos3D
    rightKnee = keypoints[right knee].Pos3D
    rightAnkle = keypoints[right ankle].Pos3D

    leftHip = keypoints[left hip].Pos3D
    leftKnee = keypoints[left knee].Pos3D
    leftAnkle = keypoints[left ankle].Pos3D

    rightShoulder = keypoints[right shoulder].Pos3D
    leftShoulder = keypoints[left shoulder].Pos3D
    upperAbdomen = keypoints[upper abdomen].Pos3D


    // Calculate bilateral knee flexion in the sagittal Y-Z plane

    rightThigh =
        rightHip - rightKnee

    rightShank =
        rightAnkle - rightKnee

    projectedRightThigh =
        vector(0, rightThigh.y, rightThigh.z)

    projectedRightShank =
        vector(0, rightShank.y, rightShank.z)

    rightKneeFlexion =
        180 degrees
        - ANGLE(projectedRightThigh, projectedRightShank)
```

```
    leftThigh =
        leftHip - leftKnee

    leftShank =
        leftAnkle - leftKnee

    projectedLeftThigh =
        vector(0, leftThigh.y, leftThigh.z)

    projectedLeftShank =
        vector(0, leftShank.y, leftShank.z)

    leftKneeFlexion =
        180 degrees
        - ANGLE(projectedLeftThigh, projectedLeftShank)


    // Both knees must reach the target

    currentSquatDepth =
        MINIMUM(rightKneeFlexion, leftKneeFlexion)


    // Save posture measurements at the deepest squat position

    IF currentSquatDepth > peakSquatDepth

        peakSquatDepth = currentSquatDepth

        trunkVector =
            ((rightShoulder + leftShoulder) / 2)
            - upperAbdomen

        trunkFlexionAtPeak =
            ROUND(ANGLE(trunkVector, world vertical))
            + trunkAngleOffset


        rightValgusAtPeak =
            rightKnee.x + valgusThreshold
            < rightAnkle.x

        leftValgusAtPeak =
            leftKnee.x - valgusThreshold
            > leftAnkle.x

    END IF

END UPDATE


FUNCTION EvaluateSquat

    assessingSquat = FALSE
    disable all feedback images


    // Feedback priority 1: knee valgus

    IF rightValgusAtPeak == TRUE
       AND leftValgusAtPeak == TRUE
```

```
    display rightValgusImage
    display leftValgusImage

    DisplayFeedback(
        "Externally rotate both knees",
        feedbackDuration
    )


ELSE IF rightValgusAtPeak == TRUE

    display rightValgusImage

    DisplayFeedback(
        "Externally rotate your right knee",
        feedbackDuration
    )


ELSE IF leftValgusAtPeak == TRUE

    display leftValgusImage

    DisplayFeedback(
        "Externally rotate your left knee",
        feedbackDuration
    )


// Feedback priority 2: insufficient trunk flexion

ELSE IF trunkFlexionAtPeak <= minimumTrunkFlexion

    display trunkFlexionImage

    DisplayFeedback(
        "Increase your trunk flexion",
        feedbackDuration
    )


// Feedback priority 3: insufficient squat depth

ELSE IF peakSquatDepth < targetSquatDepth

    display squatDepthImage

    DisplayFeedback(
        "Squat deeper",
        feedbackDuration
    )


// No detected error

ELSE

    play excellentAudio

    DisplayFeedback(
```

```
            "Excellent",
            feedbackDuration
        )

    END IF

END FUNCTION


FUNCTION DisplayFeedback(message, duration)
    feedbackText = message
    enable feedbackText
    feedbackTimer = duration
END FUNCTION


FUNCTION DisableAllFeedback
    disable rightValgusImage
    disable leftValgusImage
    disable trunkFlexionImage
    disable squatDepthImage
    disable feedbackText
END FUNCTION
```

S2 Interview Outline for Physiotherapists

About the Physiotherapists

How long have you been a therapist?

What patients do you primarily treat?

What is the estimated total number of patients you deal with?

What is the estimated average number of patients you deal with per year?

What qualifications do you hold? PT, OT, or CEP?

About the System

Overall system experience

1. After trying the complete workflow and all four components, what do you consider to be the system's main strengths and limitations?
2. How would you evaluate the feedback across the system?
3. What do you think about using both pain and movement accuracy to adapt the next set?
4. What is your view of the RGB camera and the baseline measured during the Learning Phase?

Fruit Pickup

What are the strengths and limitations of Fruit Pickup?

Gold Miner

What are the strengths and limitations of Gold Miner?

Hole in the Wall

What are the strengths and limitations of Hole in the Wall?

Gait Retraining FPA

What are the strengths and limitations of the toe-in/toe-out gait-retraining component?

S3 De-identified Questionnaire Responses from One Participant

1. Adapted Technology Acceptance Model (TAM) Questionnaire

Perceived usefulness

Questions	Strongly disagree				Strongly agree
Using this exercise management system can help my patients with KOA complete rehabilitation training more quickly.	1	2	3	**4**	5
Using this exercise management system cannot improve my patients' rehabilitation outcomes.	**1**	2	3	4	5
Using this exercise management system can improve the efficiency of my patients' rehabilitation training.	1	2	3	**4**	5
Using this exercise management system cannot enhance the effectiveness of my patients' rehabilitation training.	**1**	2	3	4	5
Using this exercise management system can make my patients' rehabilitation training easier.	1	2	3	4	**5**
I think this exercise management system is not helpful for my patients' rehabilitation training.	**1**	2	3	4	5

Perceived ease of use

Questions	Strongly disagree				Strongly agree
Learning to operate this exercise management system would not be easy for me.	**1**	2	3	4	5
I think it would be easy to use this exercise management system for patient rehabilitation training and management.	**1**	2	3	4	5
My interaction with this exercise management system would be clear and understandable.	1	2	3	4	**5**
I think this exercise management system would be inflexible to interact with.	**1**	2	3	4	5
It would not be easy for me to become skillful at using this exercise management system.	**1**	2	3	4	5
I think this exercise management system would be easy to use.	1	2	3	4	**5**

Attitude

Questions	Strongly disagree				Strongly agree
Using this exercise management system for patient rehabilitation training would be a good idea.	1	2	3	**4**	5
Using this exercise management system in patient rehabilitation training and management would be unpleasant.	**1**	2	3	4	5
Using this exercise management system would be beneficial for my patients' rehabilitation training.	1	2	3	**4**	5

Intention to use

Questions	Strongly disagree				Strongly agree
If this exercise management system were available, I would intend to recommend it for my patients' daily use.	1	2	3	4	5
I intend to use this exercise management system frequently for my patients' rehabilitation training as needed.	1	2	3	4	5
I do not intend to use this exercise management system frequently in patient rehabilitation training.	1	2	3	4	5
If possible, I would not use this exercise management system in patient rehabilitation training.	1	2	3	4	5
If possible, I would use this exercise management system for different tasks (clinical or non-clinical).	1	2	3	4	5
If possible, I would frequently use this exercise management system for patient rehabilitation training.	1	2	3	4	5

2. System Usability Scale (SUS) Questionnaire

Statement	Strongly disagree	Disagree	Neutral	Agree	Strongly agree
I think that I would like to use this system frequently.	1	2	3	4	5
I found the system unnecessarily complex.	1	2	3	4	5
I thought the system was easy to use.	1	2	3	4	5
I think that I would need the support of a technical person to be able to use this system.	1	2	3	4	5
I found the various functions in this system were well integrated.	1	2	3	4	5
I thought there was too much inconsistency in this system.	1	2	3	4	5
I imagine that most people would learn to use this system very quickly.	1	2	3	4	5
I found the system very cumbersome to use.	1	2	3	4	5
I felt very confident using the system.	1	2	3	4	5
I needed to learn a lot before I could use this system proficiently.	1	2	3	4	5
